# An osteobiography of a celebrity chimpanzee reflects the changing roles of modern zoos

**DOI:** 10.1038/s41598-025-88597-x

**Published:** 2025-03-12

**Authors:** David M. Cooper, Blessing Chidimuro, Stuart Black, Olivia Davis, Phillipa Dobbs, Gaia G. Mortier, Felix Sadebeck, Tobias Schwarz, Riley Smallman, Naomi Sykes, Juliette Waterman, Andrew C. Kitchener

**Affiliations:** 1 Department of Natural Sciences, National Museums Scotland, Edinburgh, EH1 1JF UK; 2 Department of Archaeology, University of Exeter, Exeter, UK; 3 Department of Geography and Environmental Science, University of Reading, Whiteknights, Wager Building, Reading, RG6 6EJ UK; 4 Veterinary Department, Twycross Zoo, Burton Road, Atherstone, Warwickshire, CV9 3PX UK; 5 Royal (Dick) School of Veterinary Studies and Roslin Institute, The University of Edinburgh, Roslin, UK

**Keywords:** Osteobiography, Zoo, Chimpanzee, Isotope analysis, Geometric morphometrics, Animal Welfare, Biological techniques, Chemical biology, Physiology, Zoology, Environmental social sciences

## Abstract

The role of zoos has shifted markedly from their origins in the nineteenth century to the present day. Long-lived and charismatic individual animals have been central to zoological collections, as priorities have changed from a primary focus on entertainment to a greater emphasis on education, conservation, research and welfare. Here, we construct the osteobiography of a celebrity chimpanzee to highlight the changing practices of modern zoos over a near half-century timescale. We applied archival, biogeochemical, pathological, and morphometric analyses to the skeleton and tissues of Choppers, a captive female western chimpanzee (*Pan troglodytes verus*), who featured in the PG Tips television advertisements in the United Kingdom in the 1970s. We show the efficacy of the osteobiographical method in revealing Choppers’ life events, including her capture from the wild, dietary transitions over decadal timescales, and her declining health in old age. These direct analytical techniques applied to her physical remains allow for a more in-depth understanding of her life history than from archival material alone. By using a mixed-method, in-depth assessment of a single captive chimpanzee, we provide a rich context for analysing the impact of the changing roles of zoos on captive animals during the late 20th and 21st centuries.

**Supplementary Information:**

The online version contains supplementary material available at 10.1038/s41598-025-88597-x.

## Introduction

Modern zoos are centres of education, research, conservation and entertainment^1–4^, yet the relative importance of these tenets, which were established in the 1920s^5^, has shifted considerably through the late 20th and 21st centuries. Keeping wild animals in captivity was widespread in Europe from the sixteenth century as a symbol of wealth and prestige, as exotic animals became available with the expansion of global trade routes and colonisation^6^. Entertainment and scientific curiosity were key draws of early zoos, which transitioned from private menageries into public spaces from the early nineteenth century. Zoos were historically centres for the exploitation of nature, but only became focussed on conservation and education (that extended to wider audiences beyond basic taxonomic classification) from the 1960s^6^. These transitions of priorities are evident through the lives of great apes in captivity, whose close affinity with humans has often led to their own celebrity status: Lady Jane (Jenny) the orangutan (*Pongo pygmaeus*), who was taken from the wild in Borneo and brought to London Zoo, was met by Queen Victoria in 1842 who stated:

*“The Orang-Outang is too wonderful preparing and drinking his tea*,* doing everything by word of command. He is frightful and painfully and disagreeably human.”*^7^.

Before her premature death at about five years old, Jenny was visited by Charles Darwin, whose observational notes on Jenny’s behaviour and emotions formed part of his arguments that the difference between humans and animals was one of degree and not of kind^8^. In 1986 Jambo, a silverback western lowland gorilla (*Gorilla gorilla gorilla*) at Jersey Zoo, gained celebrity status by trying to comfort a five-year-old boy who fell into the gorilla enclosure and lost consciousness^9^. This story was echoed in 1996 when Binti Jua, a female western lowland gorilla at Brookfield Zoo, became famous after cradling an unconscious child who fell into her enclosure^102^. These examples are contrasted with the shooting of another gorilla, Harambe, at Cincinnati Zoo in 2016, which sparked international outrage and who was personified online through social media, calling into question the role of zoos in modern society^10^. Owing to the longevity of great apes, individuals have been subject to vast changes in the human-animal relationship within zoos over half-century timescales. Osteobiography is a methodology widely used to understand humans from the past^11–17^ and is infrequently applied to animals^18–22^. Here we create a comprehensive osteobiography to describe the life of Choppers, a celebrity western chimpanzee (*Pan troglodytes verus*). In doing so, we understand the changing mission of modern zoos from the perspective of one of their residents, who lived through vast changes in the priorities of zoos, in public perceptions, and in standards of animal welfare. Born in c.1970, Choppers was famous for her role as Ada Lott in the PG Tips television advertisements in the United Kingdom during the 1970s and was euthanased on health and welfare grounds at Twycross Zoo in England in 2016.

## Early life in the wild and capture

**Fig. 1:**
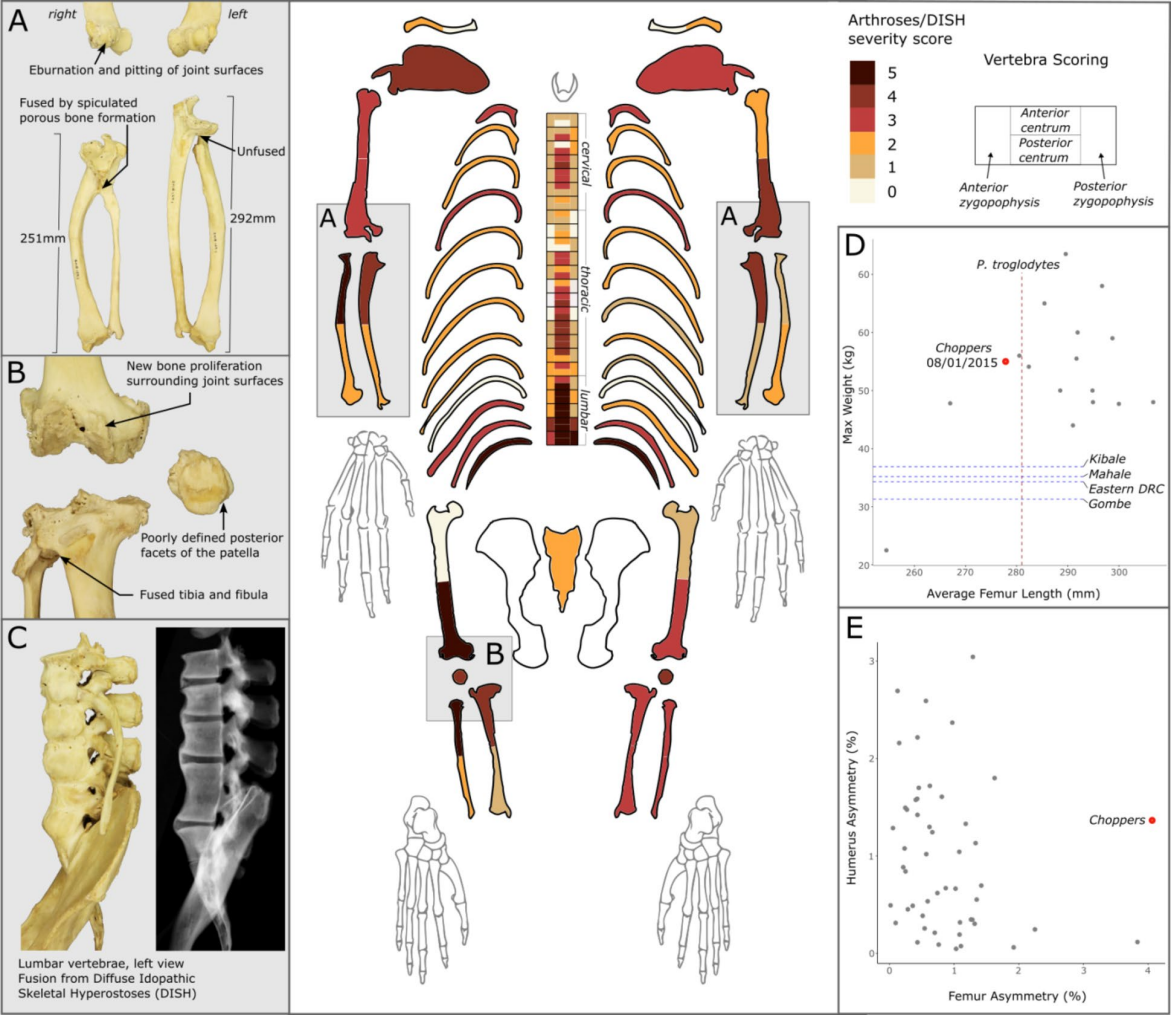
Postcranial pathologies and metric analysis of Choppers. Arthroses/Diffuse Idopathic Skeletal Hyperostosis (DISH) severity is presented visually (**main**). Choppers’ fused and severely shortened right radius and ulna have higher arthrosis scores than her left side, and are an indication of injuries likely sustained during her capture from the wild (**A**). Similarly, Choppers’ right knee, injured during her capture from the wild, exhibits greater arthroses than her left knee (**B**). The extent of DISH on Choppers’ lumbar vertebrae and pelvis is visualised (**C**), which highlights smooth osteophyte formation over the anterior ligament running over the vertebral bodies, and separation between the vertebrae, which are occupied by vertebral discs. Choppers’ maximum weight was typical of captive female chimpanzees (*n* = 18), yet her femur length was shorter than average. Her weight and that of other captive chimpanzees was typically greater than that of wild female chimpanzees (blue lines), but her femur length was below average (red line) (**D**). Choppers had considerably greater asymmetry between her left and right femora than in other captive chimpanzees (*n* = 53), as her right femur was ~ 4% shorter than her left, likely as a result of her injured knee joint. Whilst her right radius and ulna were ~ 14% shorter than her left, this did not lead to exaggerated asymmetry between her left and right humeri (**E**).

Osteometric, geochemical and pathological analyses were carried out on Choppers’ skeleton, which is in the collections of National Museums Scotland in Edinburgh (register no. NMS.Z.2018.129.1), in order to gain insights into her life from infancy to old age. These analyses supplement archival information, available due to both her celebrity status and because she lived in a modern zoological collection, where information on husbandry is routinely recorded on the international Species 360: ZIMS (Zoological Information Management System)^23^. Choppers was a western chimpanzee who was born in the wild in Sierra Leone between 1969 and 1970^23^. She was taken from the wild by poachers when she was about six weeks old, and it is suggested that she was shot in her right arm during capture and injured in her knee^24^. This is evident from a remodelled malunion fracture to the proximal shaft of both the right radius and ulna, with both shafts deformed, and shortened by ~ 14% in comparison with the left side (Fig. 1). Additionally, her right femur is ~ 4% shorter than her left one. Her mother would likely have been shot both for bushmeat and to enable Choppers’ removal and sale as a pet, thus her probable shooting injuries were incidental. It is also likely that many, if not all, of her social group were killed in her capture (e.g., see^25^). The trauma inflicted on Choppers during her capture affected her physically for the rest of her life, as in addition to the debilitating shortening of her forearm, her right elbow and left knee joints were subject to considerable arthropathy in comparison with her other long bones (Fig. 1), and this caused her pain and difficulty in movement during her later years^23^. These injuries would have affected her quadrupedal gait, and are likely the cause of her asymmetric pelvis and misalignment in some of the vertebral zygapophyses (thoracics 11, 12, lumbar 1). Choppers was purportedly rescued from the poachers by an aid worker, Diane Locke, who raised her like a human infant in Sierra Leone^24,26,27^. She was likely consuming powdered milk before human weaning age (18–24 months) and a mixed terrestrial diet, including local pap or fufu, from ~ 4 months old^28^.

### Movement from Sierra Leone to Twycross Zoo

**Fig. 2:**
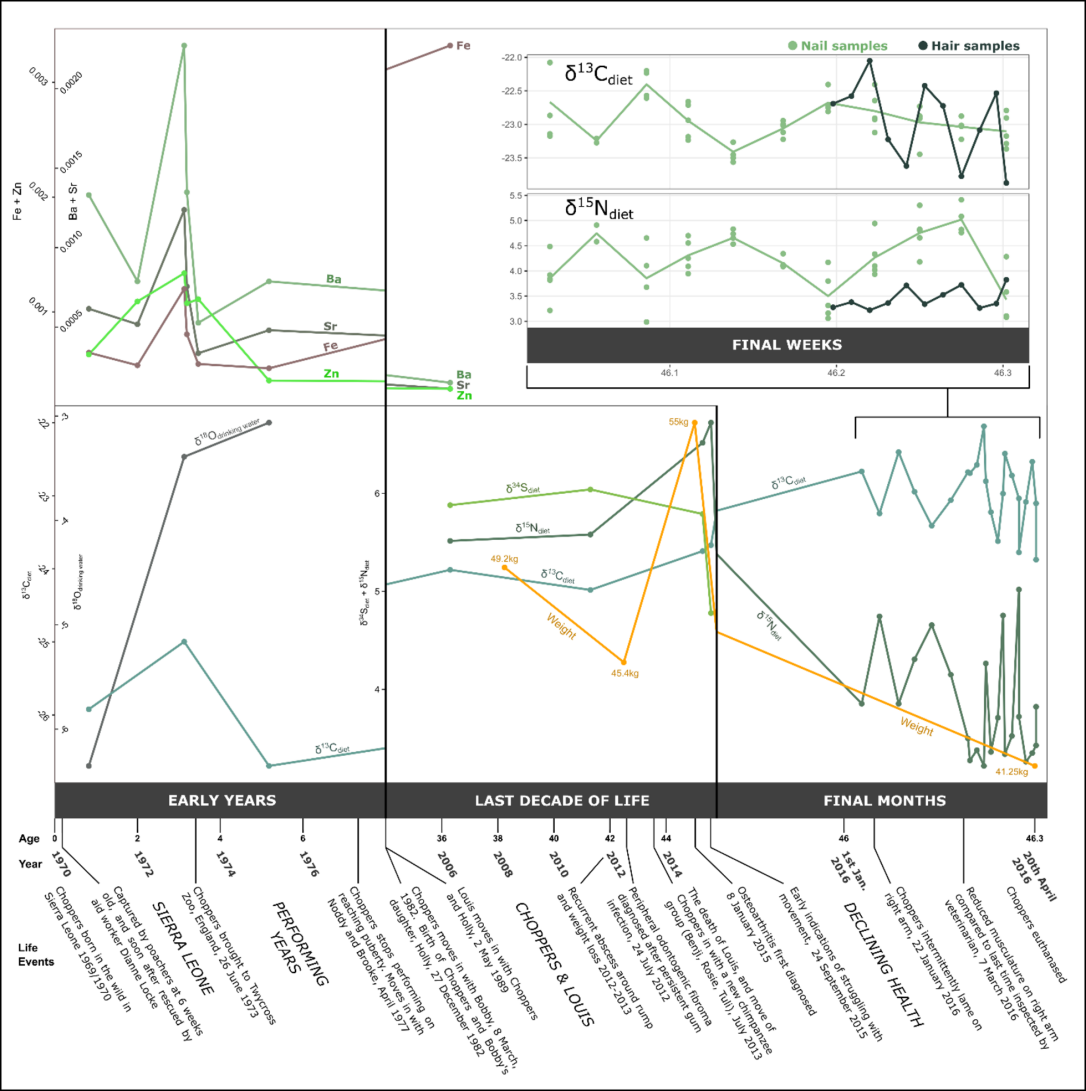
Trace element (Ba, Sr, Fe, Sn) and isotopic (^18^o_drinkingwater,_^13^c_diet,_^15^n_diet,_^34^s_diet_) changes in choppers’ tissues, taken from her tooth enamel (early years), femur (last decade of life), nail, and hair (final months and final weeks). trace element values are the mol/mol ratio with calcium. offsets applied to the isotope ratio values are presented within the supplementary information. results indicate a shift in soil geochemistry (trace elements),drinking water (^18^o_drinkingwater_),and diet (^13^c_diet_) when choppers was moved from sierra leone to twycross zoo in the uk. her diet shifted considerably from her performing years to her later life as indicated by ^13^c_diet_values. here, we include her changing weight in later life,which tracks with changes to her overall health^23^, and with declines in ^15^n_diet_values.

**Fig. 3:**
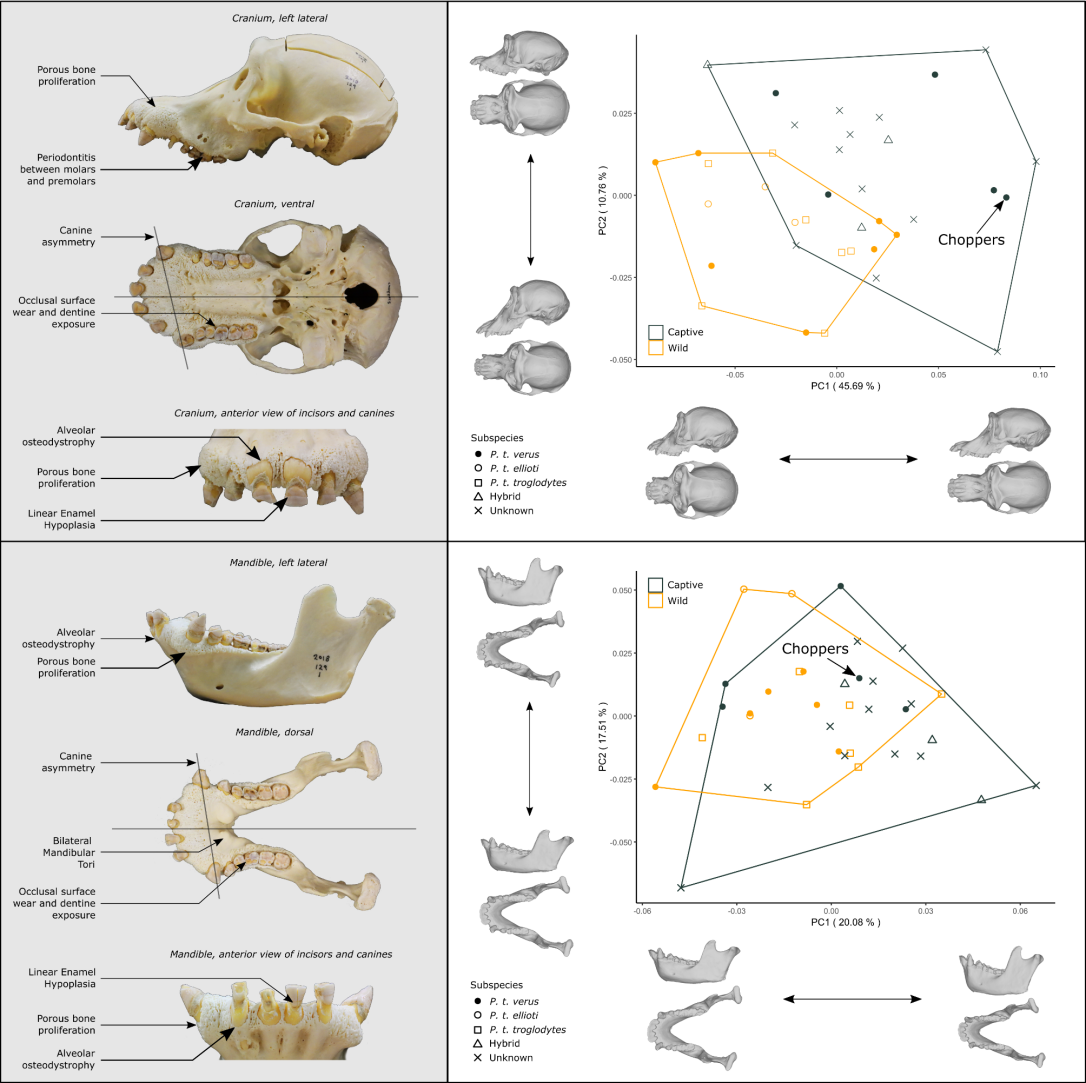
Cranio-mandibular and dental pathologies (left) and geometric morphometric analysis (right) of Choppers. The shape of Choppers’ skull and mandible has been compared with other female chimpanzees. This sample included prime (13–30 years) and old (30 + years) adults from both captivity and the wild. Western (P. t. verus), Nigeria-Cameroon (P. t. ellioti), Central (P. t. troglodytes), hybrids and chimpanzees of unknown subspecies were included within the analysis. Like other captive chimpanzees, Choppers has an elongated rostrum in comparison to those of wild chimpanzees, which in Choppers is likely exacerbated by increased porous bone proliferation. The shape of her mandible appears typical given the comparative sample of captive and wild chimpanzees.

Choppers remained in Sierra Leone until she was three to four years old, at which point she was sent to the newly opened Twycross Zoo, in the United Kingdom, under the care of Molly Badham and Nathalie Evans, arriving on 26th June 1973^24,27^. This is evident from trace element and isotopic analyses of Choppers’ tooth enamel, which indicate a distinct geographical (^18^O_DrinkingWater_) and dietary (^13^C_diet_) shift between the ages of three and four (Fig. 2). ^13^C_diet_values indicate that Choppers regularly consumed fruit from an early age, which is typical of the wild diets of infant chimpanzees, and of captive chimpanzees in the 1970s^29,30^. The reduction in 13C_diet_ values from Sierra Leone to Twycross Zoo likely reflects a decrease of C_4_plants (e.g., cassava, yams) in her diet^31^. The trace elements Ba, Sr, Zn and Fe corroborate this period of fluctuation in Choppers’ diet and location. High Sr and Ba can indicate a highly vegetarian (or low meat) diet^32–34^, but the high Sr and Ba levels correlate with an increase in Fe and Zn, which can indicate high protein or meat diet^34,35^. Therefore, it is likely that in addition to dietary changes during this time, soil chemistry from different locations during Choppers’ movements has impacted trace element signatures. Her incisors show Linear Enamel Hypoplasia (LEH) (Fig. 3), an interruption in the development of enamel in teeth as a result of physiological, nutritional and/or psychological stress during development^36^, which likely relates to these years of dietary turbulence^103^, although we note that this is also common in wild chimpanzees^37,38^. Whilst Twycross was a leading centre for captive primate care in the UK, Choppers’ acquisition was underpinned by elements of exploitation. Molly Badham, co-founder of Twycross, states about chimpanzees in the 1970s:

*“If we were to continue with our tea parties and any other public appearances*,* we knew that we would have to buy some new young chimps to take over from our old-stagers. They should preferably be under the age of twelve months if we were to be sure they would adapt to living away from the family group. For once chimps have become accustomed to living in groups of their own kind and have learned to depend on each other*,* they never truly learn to depend on humans in the same way and will never trust them.”*^26^.

Twycross Zoo and the Brooke Bond Tea Company used chimpanzees for supermarket promotion and television advertisements for the PG Tips tea brand from 1963^26^, with the chimps acting as humans, drinking tea, and dubbed over with human voices. Choppers played Ada Lott, the grandmother character (despite her young age of between four and seven years old) in the later TV adverts during the 1970s. These adverts helped PG Tips become the market leader of tea in Britain for 35 years^39^. Her performance career was short, occurring before the onset of puberty, and Choppers probably retired at around the age of six or seven years old^40^. In part this is due to behavioural change as adult chimpanzees become less predictable, but also as a result of human perceptions of the cuteness of adult chimpanzees compared to infants:

“*Once they grow big and develop huge arms and chests and weigh up to 100lb they no longer look acceptable dressed up.*”^26^.

In the late 1970s Choppers transitioned from a relatively active life with high levels of direct interaction with humans, to a sedentary life with two companion chimpanzees, Noddy and Brooke, who were also retired from the entertainment industry^23^. This was deemed a necessity due to the lack of prior interaction and habituation with non-performing chimpanzees, but would have inevitably been a less stimulating and physically smaller environment than that which could be provided with integration into a larger chimpanzee social group^26^. Choppers’ potential daily movement for much of her life would have been greatly reduced compared to that of large social groups in much larger enclosures, and when compared to the 2–4 km daily travel distances of wild chimpanzees^41^. Choppers’ diet during her performing years, and the years immediately following was instrumental in her growth, development, and health throughout life, as female chimpanzees reach adult size in captivity around the age of 11 years old^42^. The performance diets of chimpanzees at Twycross mimicked those of humans, following on from a longstanding trope in the mid-twentieth century of chimpanzees participating in tea parties, eating cake, drinking ‘tea’, and ‘apeing’ human behaviours and society^26,40,43^. This fascination with primate anthropomorphism in Northwest Europe is documented from at least the 18th century when apes were first imported from Africa and Asia^6^. The chimpanzees drank fruit juice or milk rather than tea during tea parties and advertisements^43^, and it is likely that Choppers had a predominantly fruit-based diet as indicated by ^13^C_diet_ values (Fig. 2) and feeding practices of chimpanzees at the time^26,29^. Throughout Choppers’ life values of ^13^C_diet_, ^15^N_diet_, and ^34^S are consistent with a mixed terrestrial plant and animal diet, and so Choppers would have been receiving diverse supplemental foods beyond fruit during this time, including high sugar treats, but likely also protein sources such as eggs^26^. Based on her mean femoral measurements, Choppers was smaller than average for both captive and wild female chimpanzees, yet her weight was much higher than that of wild chimpanzees, and typical of that of captive female chimpanzees (Fig. 1). This is likely a result of a positive energy balance due to greater calorific intake and lower physical activity than those of wild chimpanzees^42^, which may have been exacerbated by her injuries and the subsequent development of arthroses that reduced her mobility. Captivity is often associated with obesity^44,45^, but it is noted in her veterinary record (19/04/2012), when Choppers was 43 years old, that she had always been a lean chimpanzee, and we note that her maximum weight likely reflected a conscious effort to increase her body condition following illness^23^ (Fig. 2).

Whilst Choppers’ diet in the 1970s/1980s must have provided the calorific content and macronutrients to obtain her large adult size, her dental pathologies are indicative of mechanical deficiencies in her diet. Her canine asymmetry in her maxilla and mandible, and dental malocclusion are likely to have developed during adolescence. Mechanically softer diets have been found to cause malocclusion in humans^46^, non-human primates^47^, and other vertebrates^48^. Soft foods are prevalent in modern human diets^49^ and have been a staple of zoological feeding programmes across many taxa through the latter half of the 20th century^50,51^. Whilst wild chimpanzees consume significant quantities of fruit, they must dehusk and process hard outer materials, and must spend more time chewing and consuming tougher fruit that is of a lower calorific content than cultivated fruits for human consumption, thereby increasing the peak and cumulative mechanical stresses in the skull and mandible^52^. The elongation of Choppers’ maxilla is characteristic of captive chimpanzees^53^ (Fig. 3). Alongside her dental pathologies and associated anterior bone proliferation, her long rostrum is likely due in part to the mechanical influence of a soft diet during the development of a weaker musculo-skeletal system^53,54^.

### Late life

Choppers’ preserved remains provide us with information from her early life, through her tooth enamel, pathologies, and skeletal morphology, which formed during development. They also provide us with information towards the end of her life due to the turnover of bone and keratinous tissues, which hold isotopic signatures of diet and health, and from the development of pathologies associated with old age. A limitation of an osteobiography of a long-lived animal is that there are decades of Choppers’ adult life which cannot be accounted for until a few years before her death (Fig. 2). Choppers was re-housed with another chimpanzee, Bobby, on 8th March 1982, and together they had one daughter, Holly, born on 27th December 1982 (when Choppers was 13). Replacing Bobby, Louis (another performing chimpanzee who had played Mr Shifter in the PG Tips adverts^27,55^) had been rehoused with Choppers and Holly in 1989. Holly was later re-housed and lived at Twycross Zoo until her death on 9th November 2023^23^.

The turnover rate of femoral bone in chimpanzees is ~ 10 years^56–58^, and consequently we can infer changes to Choppers’ diet through her bone collagen in the last 5–10 years of her life. Therefore, we pick up Choppers’ adult story through her tissues in 2006 (~ 10 years before death), when Choppers was 38 and cohabiting with Louis. This period is complemented by well-documented health and veterinary records from 2010^23^ and dietary information since 2011 (see *Twycross Zoo Chimpanzee Diet Sheet 2011* within the *Supplementary Information*). Choppers’ diet in her later years consisted of commercial primate pellets, browse and vegetables, limited quantities of fruit and yoghurt, and the occasional egg. This contrasts against the diet during her early years that is presumed to have been high in fruit and sugar. The ^13^C_diet_ values from Choppers’ bone samples (signifying the last 10 years of her life) are stable, and higher than those of her early life in Sierra Leone and Twycross Zoo (Fig. 2). This reflects a prescriptive modern diet plan, and an increase in C_4_ plant intake from maize protein in primate pellets, and from flaked maize and popcorn scatter feeds.

Average life expectancy of wild chimpanzees ranges from late 30s^59^ to 40s^60^ with maximum ages of some individuals surpassing 50 years^59^. Chimpanzees exhibit an increased susceptibility to infections and bone related pathologies from their 20s and experience reproductive and cognitive decline after the age of 30^61–63^. Thus, Choppers lived ~ 17 years of her life as an elderly chimpanzee (post 30 years old) before her death at 47. She had degenerative skeletal pathologies associated with old age, but these may have been exacerbated by her traumas during infancy and subsequent mobility impairment, as well as her environment (e.g. harder substrates found in captivity^64^) and diet. Owing to thorough documentation of Choppers’ health and veterinary record since 2010, we are able to compare known and suspected health issues in life with resulting skeletal pathologies in death. Ten of her molars and premolars (81% of teeth analysed) exhibited enamel wear with resulting dentin exposure of up to 1/3 of the total occlusal surface, with two teeth (12.5%) exhibiting dentin exposure over 1/3 (see *Table S3* of the *Supplementary Information*). This level of wear is typical of wild chimpanzees^65^. She had chronic alveolar osteodystrophy and chronic low-grade periodontal disease and severe bone proliferation around her mandible and maxilla (*Fagan and Woody*,* pers. comms.*). This is consistent with persistent gum infections in the winter and spring of 2012 (four years before death). Choppers’ abnormal cranial shape has resulted partly from an unknown developmental trauma (canine asymmetry) and the extensive proliferation of alveolar bone. She was diagnosed through a biopsy as having peripheral odontogenic fibroma in July 2012^23^, but this is not reflected in her skull morphology. Choppers had bilateral mandibular tori growth (Fig. 3). Whilst this likely has a genetic component, it may be a consequence of increased mechanical stress as a result of temporomandibular dysfunction, or due to parafunctional activity such as bruxism due to psychological stress^66–68^. Mandibular tori may also form due to dietary deficiencies, or an excess of calcium supplementation^68^, both of which are unlikely given Choppers’ varied diet plan in late life (see *Twycross Zoo Chimpanzee Diet Sheet 2011* within the *Supplementary Information*).

Choppers’ long-term companion, Louis, died in 2013 and so at the age of 45 years old, Choppers had to integrate with a new group of chimpanzees. It is likely that Choppers’ hand rearing and later life spent paired with a single chimpanzee may have significantly compromised her ability to socialise with other chimpanzees^69^. Captive older female chimpanzees exhibit more submissive behaviours than younger chimpanzees^70^, and high-value social relationships between chimpanzees are harder to establish later in life^71^. Choppers’ introduction to Benji, Rosie and Tuli in July 2013 led to minor fighting and superficial injuries, with some bullying occurring from Tuli for several months^23^.

Choppers’ skeleton shows multiple indications of diffuse idiopathic skeletal hyperostosis (DISH), and extensive arthritic degeneration in most joints of the long bones (Fig. 1). DISH is typically manifested as smooth ‘candle-wax’ osteophyte formation surrounding the vertebral bodies due to ossification of the anterior ligament, and in Choppers the three caudal-most vertebrae and the sacrum are fused together. The cause of DISH is unknown and is usually asymptomatic, but it has been associated with rich diets and excess body fat in humans, as well as being associated with coronary heart disease, diabetes mellitus, and inflammatory bowel disease^72^. Whether these factors are causal or correlated, and whether DISH causes pain, is unclear. However, DISH, alongside extensive arthroses within the lumbar region would have limited Choppers’ mobility. Visual inspection of her movement indicated arthritis in Choppers in 2013 (only 2.5 years from her death)^23^. In her later life it was apparent that arthritis (diagnosed in 2015, but likely to have manifested much earlier)^23^ was significantly affecting her movement, affecting her hind legs (right knee injured during her capture) on occasion (22/01/2016), and often affecting her injured right arm, leading to significant muscle wastage on this limb^23^.

## Final weeks and death

Using hair and samples through sectioned nail, we gained insight into the final four months of Choppers’ life (Fig. 2). Choppers exhibited marked weight loss of ~ 25% during her last year of life as her health deteriorated^23^. Such physiological stress can be associated with an increase in *δ*^15^N in body tissues due to tissue catabolism^73^, but Choppers’ tissues markedly decreased in *δ*^15^N in her final year. We speculate that this change is due to Choppers consuming less high-protein food (nuts, yoghurt, eggs, etc.) during her final months, even if these were available in her diet. Choppers, the last surviving chimpanzee from the PG Tips tea commercials, was euthanased on 20th April 2016 following observed jaundice, a persistent cough, and lethargy, and in light of severe weight loss and behavioural change^23^. A post-mortem report indicated that Choppers suffered from chronic hepatitis and cardiomyopathy. She also had yersiniosis (*Yersinia enterocolitica*), which was the first known case in a captive chimpanzee^23^.

## Discussion

Osteobiography as a tool has been applied to human lives in the past, most often of people beyond contemporary human memory, where biographical archival material is limited^11–17^. There is an increasing application of the tool to understand the lives of modern animals, whose individual histories are poorly understood due to their lack of interaction with humans during life^21,22^. Choppers lived during a period of recent history within a well-documented zoo, and her celebrity status means that rich archival information about her life has been readily available, and yet as a chimpanzee, first-hand accounts of her life are not possible. Animal voices and experiences are obscured by a lack of human understanding and human representation of what we *think*the animal experience was^74^, and whilst here we describe Choppers from a human point of view, we provide an analytical perspective in death, creating a richer context to first-hand human accounts of her life: We know that Choppers was taken from the wild, but here we can visualise the sharp dietary and geographical changes that ensued directly through her body - effects which she carried through life. We have visualised and scored the extent of bone eburnation and osteoarthritis in Choppers’ right elbow and knee, which trigger further empathy to accounts of her injuries during capture and mobility difficulties in later life. We have categorised the extent of DISH on her spine, which lends additional evidence to her reduced mobility that would be difficult to appreciate through observation during her life alone. Choppers highlights the efficacy of osteobiography in understanding the formative years of an individual (encapsulated through developmental plasticity, injury, and the formation of tooth enamel), and of the years before her death (through age-related skeletal pathologies and the chemistry of tissues which turn over throughout life). By analysing different tissues, such as tooth enamel from teeth that erupt at different times, bone, hair, and nail, we can obtain snapshots of her diet and physiology at different stages of her life from infancy to very old age. However, the long period that Choppers lived through from the 1980s until the 2010s is largely undocumented and without trace in her physical remains.

Choppers’ cranial and postcranial morphology, which differ from those of her wild conspecifics, creates a story not just of herself, but for captive chimpanzees from the late 20th and 21st centuries, who have experienced similar shifts in environment, husbandry, and human-animal relationships. Her story is representative of the PG Tips chimpanzees, but also of great apes in captivity globally that have experienced shifting conditions and attitudes over decadal timescales.

By the 1970s Twycross Zoo was (as it is today) a leading authority on primate care and breeding^40,75,76^, yet attitudes towards wild animals in captivity and the role of zoos have changed considerably from Choppers’ birth in 1969/1970 to her death in 2016. The origins of animal welfare and modern zoological research in Britain are found in the 18th century^77,78^, but it is apparent through her traumatic capture from the wild and use in television that Choppers represents a period, starting from the beginnings of European menageries and modern zoos, where animals were routinely extracted from the wild and entertainment was central to human relationships with wild animals. However, Choppers lived through widespread advances in zoological research, welfare, and conservation - all core tenets of modern zoos. The dietary change between her performing years in the 1970s and her life in the 2010s exemplifies this change in knowledge and husbandry (e.g., through a reduction in cultivated fruit, which is higher in simple sugars than wild fruits, and by providing a diet that better replicates the nutrient profile of wild diets^29,79^). Whilst nature conservation in Britain can be traced to the 17th century^80^, it rose to prominence in its modern form through the latter half of the 20th century^5^, and zoos were instrumental in developing Taxon Advisory Groups from the 1980s, and later Species Survival Plans (USA)/European Endangered Species Programmes (Europe) and international/regional studbooks for better conservation management^81^. The introduction of the Convention on International Trade in Endangered Species (CITES) during the mid-1970s^6^ made the removal of animals from the wild more difficult and less common, thereby further promoting breeding programmes within captivity. Choppers’ extraction from the wild in 1969/1970 resulted in lifelong physical injuries and likely the death of multiple wild chimpanzees. She was rescued by Dianne Locke and later Twycross Zoo on justifiable welfare grounds, but which involved further exploitation of Choppers in television adverts in the 1970s, which would be unacceptable today. Whilst direct human interactions and performing may have been enriching for the young chimpanzees involved, it was temporary in nature, and the use of chimpanzees in commercials may have actively undermined conservation goals by distorting public perceptions of wild animals^82^. Indeed, the withdrawal of the high levels of stimulation during these early years of performance would likely have been highly traumatic over several decades. Despite this, through changes to zoo practices over the last 40 years, which has resulted in a shift in their core priorities, Choppers died as an ambassador for her species in captivity, and not as an ageing entertainer ‘apeing’ human behaviour. DISH, dental pathologies, and extensive arthroses (which are widespread beyond Choppers’ injured limbs) are all likely in-part to be related to her old age, and so Choppers’ later life raises new questions regarding the management of ageing zoo animals made prevalent by husbandry and veterinary advances^44^.

Whilst Choppers’ story tells us about changing zoo practices through time in Britain, there is considerable global variation in zoo scrutiny, management and welfare today. Despite regional and global accreditation of zoos and improved regulation, the illegal trafficking of chimpanzees and other primates into private collections and disreputable zoos continues^83^. Choppers’ story, as told by her remains and archival records, are testament to the many thousands of chimpanzees that were forcibly extracted from the wild - for zoos, circuses, laboratories and private collections - and similar stories will continue to be revealed as modern chimpanzee populations are exploited today and in the future. Choppers was not an unusual chimpanzee, but her story is an individual one, which resonates with human attitudes towards wildlife, zoos, entertainment, welfare and quality of life.

## Materials and methods

Choppers’ skeleton, nails, and hair were prepared at National Museums Scotland, where she is registered as part of the research collections (register no. NMS.Z.2018.129.1).

### Pathological analysis

High-resolution photographs were taken of Choppers’ dentition and jaws to allow detailed assessment of oral pathologies. These photographs were reviewed by veterinary dentists Dr David Fagan, The Colyer Institute; Dr Allison Woody, San Diego Zoo. The percentage of dentin exposure on Choppers’ molars and premolars was calculated from photographs using ImageJ^84^ software. *Estimates of total occlusal surface and total exposed dentin were made where postmortem tooth damage had occurred in small discrete locations e.g. to the edge of the occlusal surface where enamel had been removed for isotopic analysis.*

Choppers’ skeleton was examined for skeletal pathologies, which included the following broad categories:

1. Traumas, including healed fractures.
2. Osteoarthroses, mostly of the long bones, where osteophytes and eburnation are apparent.
3. Spondyloarthroses, including the presence of osteophytes on the vertebrae and also including Diffuse Idiopathic Skeletal Hyperostosis (DISH). It may be difficult to be certain whether arthroses have developed because of non-inflammatory or inflammatory causes so that for the purposes of this analysis, no further analysis was attempted. In addition to a description of the pathologies, the degree of development of osteophytes on different parts of Choppers’ skeleton were recorded following^85^. Scores range from 0 (no osteophytes) to five (fusion of joints by osteophytes). Arthroses, spondyloarthroses and DISH were scored on left and right sides separately for all long bones, vertebrae and the sacrum/pelvis.

## Morphometric analysis

We obtained 3D scans of the skulls and mandibles of 37 adult female chimpanzees (20 captive, 17 wild). Western chimpanzee specimens (*P. t. verus*) were supplemented with Nigeria-Cameroon (*P. t. ellioti*), Central (*P. t. troglodytes*), hybrid, and chimpanzees of unknown subspecies to create a larger sample size. Scans were obtained using an EinScan H structured light surface scanner (accuracy: ±0.05 mm), and through MorphoSource (www.morphosource.org). We used 3D geometric morphometrics to characterise the size and shape of both the skull and mandible (see *Supplementary Information*for specimen list and landmarking protocol). Landmarks were placed using 3D Slicer^86^ and imported into the R environment^87^ for analysis using the SlicerMorph package^88^. Procrustes superimposition and principal component analysis was performed in the geomorph package^89^ in R. The results of this analysis (Fig. 3) are provided within the *Supplementary Information*in relation to specimen age class (prime adult: 13–30 years old, and old adult 30 + years old). The greatest length of the left and right femur and humerus were taken from Choppers and 51 captive adult chimpanzees using digital dial calipers (accuracy: ±0.1 mm). Published average wild female western chimpanzee femur length^90^ was used for comparison. Body weights from Choppers, and maximum body weight from 20 captive adult female chimpanzees were obtained from Species 360: ZIMS (Zoological Information Management System)^23^ and compared with published average body weights from wild chimpanzee populations^91^.

## Isotopic and trace element analysis

By combining stable isotope and trace element analyses between different tissue types and structures, we reconstructed Choppers’ diet over time (Table 1).

**Table 1:** Elements of choppers’ remains utilised for isotopic and trace element analysis, with associated ages of deposition.

Element	Structure	Age range	Reference
Nail	Keratin	3 mm per month from date of death	^98^
Hair	Keratin	1 cm per month from date of death	^100^
Rib	Bone	5–10 years from date of death	^56,58,97^
Tibia/femur	Bone	10 years from date of death	^56,58^
Skull	Bone	Assumed depositional time range of rib and long bones	
Candlewax bone (DISH)	Bone	Reflects onset of pathology	
1st molar	Enamel	0–1.65 years from birth	^101^
2nd molar	Enamel	1.34–5.05 years from birth	^101^
3rd molar	Enamel	3.11–7.25 years from birth	^101^
4th pre-molar	Enamel	1.48–4.78 years from birth	^101^
Canine	Enamel	0.49–6.45 years from birth	^101^
Central incisor	Enamel	0.5–3.5 years from birth	^101^

Tissues that grow incrementally, such as teeth, nails and hair, are ideal for studying diet at various stages in life as they record the stable isotope values at the time of tissue formation^92^. Tooth enamel does not remodel once formed and therefore carbon and oxygen incorporated into the enamel hydroxyapatite structure are retained throughout life, serving as a record of diet during enamel mineralisation^93^. The enamel of different teeth forms at different times during development (Table 1), giving an almost annual insight into the first seven years of Choppers’ life.

Bone remodels constantly so that stable isotope analysis of bone reveals the average diet over varying periods of time^94–96^. The histological development of bone is similar between humans and chimpanzees^57^. For example, femoral bone reflects an individual’s diet over approximately the last 10 years of life^56,58^, whereas ribs have faster turnover rates and represent diet from within a period of five to 10 years prior to death^56,58,97^. Sections of hair and nail are representative of diet in the weeks and months prior to death. Assuming a human nail growth rate of 3 mm per month^98^, Choppers’ 11-mm-long nail is representative of the food she ate in the last four months of her life. Primate and human hair grow at comparable rates^99^, and strands of Choppers’ hair provide data on the last two weeks of her life. The methodological procedures for isotopic and trace element analyses are described in detail within the *Supplementary Information*.

## Electronic supplementary material

Below is the link to the electronic supplementary material.

Supplementary Material 1

## Data Availability

Data are available within the supplementary information.
