# Supplementary material for: An osteobiography of a celebrity chimpanzee reflects the changing roles of modern zoos
Source: Sci Rep. 2025 Mar 12;15:7190. doi: 10.1038/s41598-025-88597-x (PMC11904210; doi:10.1038/s41598-025-88597-x)
Supplement: Supplementary file 1 — Supplementary Material 1 [file 41598_2025_88597_MOESM1_ESM.pdf]

# Supplementary Information

## An osteobiography of a celebrity chimpanzee reflects the changing roles of modern zoos.

David M. Cooper [1,2*], Blessing Chidimuro [2,3], Stuart Black [3], Olivia Davis [2], Phillipa Dobbs [4], Gaia G. Mortier [3], Felix Sadebeck [2], Tobias Schwarz [5], Riley Smallman [2], Naomi Sykes [2], Juliette Waterman [3], Andrew C. Kitchener [1,3]

[1] Department of Natural Sciences, National Museums Scotland, Edinburgh EH1 1JF, UK
[2] Department of Archaeology, University of Exeter, Exeter, UK
[3] Department of Geography and Environmental Science, University of Reading, Whiteknights, Wager Building, Reading RG6 6EJ, UK
[4] Veterinary Department, Twycross Zoo, Burton Road, Atherstone, Warwickshire, CV9 3PX, UK
[5] Royal (Dick) School of Veterinary Studies and Roslin Institute, The University of Edinburgh, Roslin, UK
*d.cooper@nms.ac.uk

## Contents

An osteobiography of a celebrity chimpanzee reflects the changing roles of modern zoos. 1
Extended Methods ..... 2
1.1 Morphometric Analysis Landmark Protocol ..... 2
1.2 3D Morphometrics Museum Accession Numbers and Metadata ..... 5
1.3 Isotopic and Trace Element Analysis ..... 6
Stable Isotope Analysis ..... 6
Trace Element Analysis ..... 8
Extended Results ..... 9
2.1 Photographs of Skeleton ..... 9
2.2 Photographs in Life ..... 21
Dentin Exposure Results ..... 22
2.3 Pathology Score Tables ..... 23
2.4 Geometric Morphometrics Results ..... 25
2.5 Linear Morphometrics Results ..... 25
2.6 Isotope and Trace Element Analysis Data ..... 27
2.7 Twycross Zoo Chimpanzee Diet Sheet (2011) ..... 29
..... 30
References ..... 38

# Extended Methods

## 1.1 Morphometric Analysis Landmark Protocol

Normal landmarks of the cranium adapted from Frost et al., (2003)[1], and of the mandible were combined with sliding semi-landmarks to represent the overall shape of each structure (*Figure S1* + *Figure S2*).

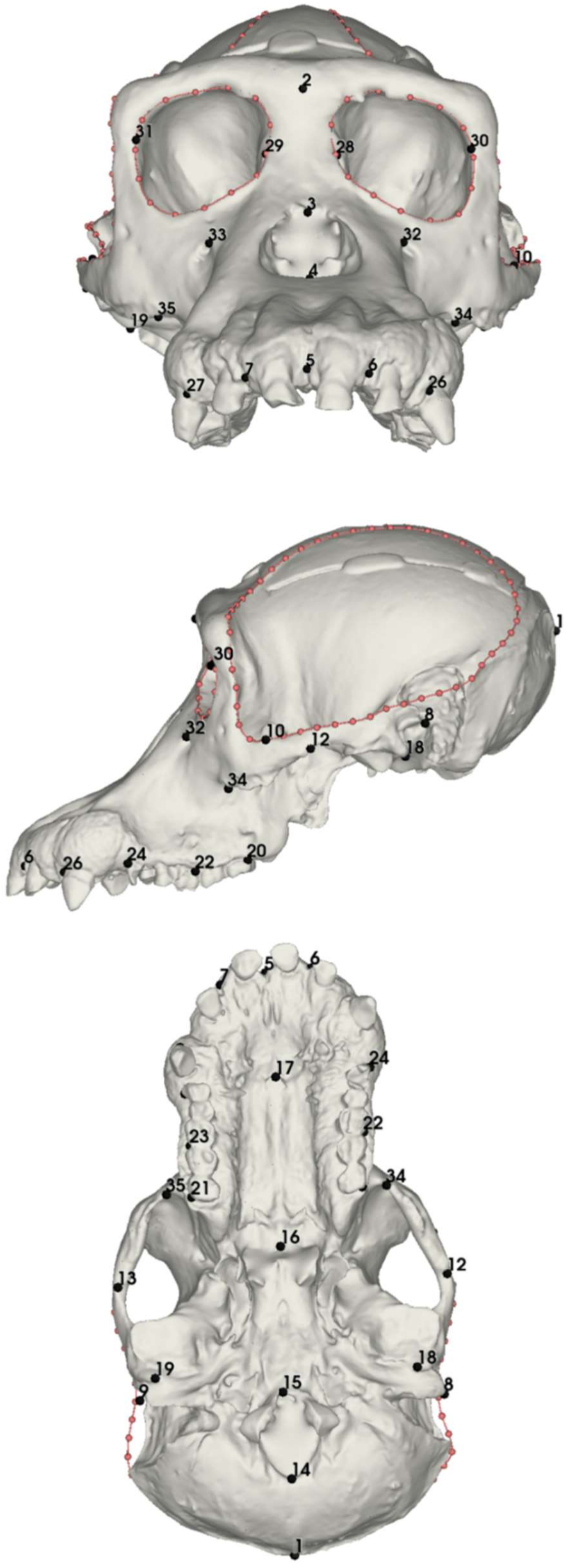

**Normal Landmarks**

1. Inion: most posterior point of the cranium, when viewed in the Frankfurt horizontal, be it on the sagittal/nuchal crest or not.

2. Glabella: As viewed in the Frankfurt horizontal.

3. Rhinion: Most anterior point in midline on nasals.

4. Nasospinale: Inferiormost midline point on piriform aperture.

5. Prosthion: Anteroinferior point on projection of premaxilla between central incisors.

6+7. Prosthion2: Antero-inferiormost point on pre maxilla, equivalent to prosthion, but between central and lateral incisors (left & right).

8+9. Auditory meatus: Most posterior point of the lateral rim of the auditory meatus when viewed in Frankfurt horizontal (left & right).

10+11. Zygo-temp superior: Superior point of zygomatico-temporal stuture on lateral face of zygomatic arch (left & right).

12+13. Zygo-temp inferior: Inferolateral point of zygomaticotemporal suture on lateral face of zygomatic arch (left & right).

14. Opisthion: Posterior most point of foramen magnum.

15. Basion: Anterior most point of foramen magnum.

16. Staphylion: Midline point on palate on line tangent to anterior most points on choanae.

17. Incisivion: Midline point at the anterior most point of the maxilla, extrapolated if broken or asymmetrical.

18+19. Postglenoid: Tip, or midpoint of area (left & right).

20+21. Posterolateral M3: Most posterolateral corner on M3 projected onto alveolar margin (left & right).

22+23. M1-2 contact: Projected laterally onto alveolar margin (left & right).

24+25. Anterolateral P3: Most anterolateral corner on P3 alveolus, projected onto alveolar margin.

26+27. Anterior Canine: Projected anteriorly onto alveolar margin (left & right).

28+29. Dacryon: Junction of frontal, lacrimal and maxilla (left & right).

30+31. Frontomalare orbitale: Where frontozygomatic suture crosses the inner orbital rim (left & right).

32+33. Infraorbital foramen: Most superior point of the rim of the largest foramen (left & right).

34+35. Zygomatic protuberance: Most anteroinferior point of the zygomatic bone.

**Sliding Semi-Landmarks (Red)**

Surrounding the temporal fossa: Tracing the superior edge of the zygomatic arch, extending around the latero-posterior margin of the termporal and frontal bone, joining the superior temporal line and superior mastoid crest (left & right).

Surrounding the orbit: Tracing the interior-anterior edge of the orbit (left & right)

*Figure S1: Normal and sliding semi-landmarks of chimpanzee crania for 3D geometric morphometric analysis. Normal landmarks have been modified from Frost et al., (2003)[1].*

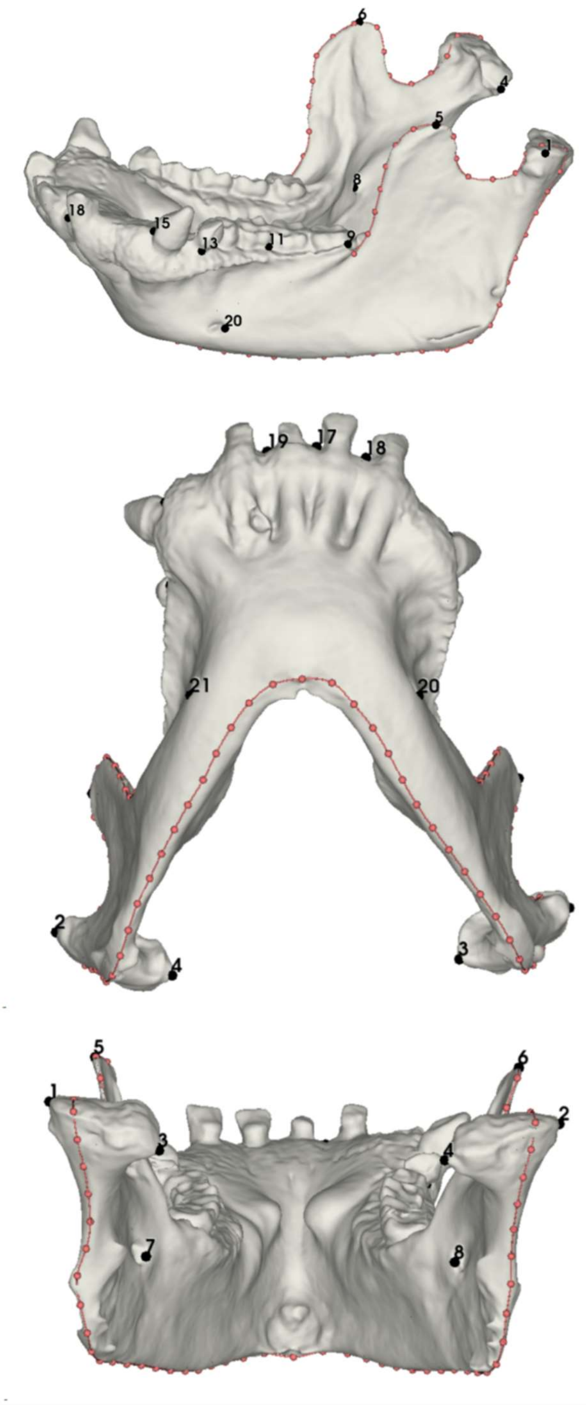

**Normal Landmarks**

1+2. Lateral condyle: Most lateral point of mandibular condyle (left & right).

3+4. Medial condyle: Most medial point of mandibular condyle (left & right).

5+6. Coronoid process: Most superior point of coronoid process, when mandible is positioned on flat surface (left & right).

7+8. Mandibular foramina: most anterior point on the rim of the mandibular foramina (left & right).

9+10. Posterolateral M3: Most posterolateral corner on M3 projected onto alveolar margin (left & right).

11+12. M1-2 contact: Projected laterally onto alveolar margin (left & right).

13+14. Anterolateral P1: Most anterolateral corner on P1 projected onto alveolar margin.

15+16. Anterior canine: Projected anteriorly onto alveolar margin (left & right).

17. Anterior midline: Anteriosuperior edge of the mandible between central incisors (left & right).

18+19. Anterior2: Anterosuperior edge of the mandible, equivalent to 'Anterior midline', but between central and lateral incisors (left & right).

20+21. Mental foramen: Most posterior point on rim of the mental foramen, when mandible is positioned on flat surface (left & right).

**Sliding Semi-Landmarks (Red)**

Originating at left anterior edge of the coronoid process, where it is level with the alveolus of the molar teeth (e.g. Normal landmark 9), extending posteriorly around the coronoid process, condyloid process and ramus of the mandible, and continuing anteriorly along the inferior edge of the mandibular body. The line continues along the right side of the mandible to terminate at the right anterior edge of the coronoid process.

*Figure S2: Normal and sliding semi-landmarks of chimpanzee mandibles for 3D geometric morphometric analysis.*

## 1.2 3D Morphometrics Museum Accession Numbers and Metadata

*Table S1: Female chimpanzees utilised in 3D geometric morphometric analyses. Specimens were obtained from the Royal College of Surgeons, England (RCS), The Smithsonian National Museum of Natural History via Morpho Source (www.morphosource.org), The Powell-Cotton Museum, and National Museums Scotland (NMS). We included adult chimpanzees during prime (13-30) and old (30+) stages of life (if known) from both captivity and the wild.*

| Collection | Register no. | Age class | Status | Subspecies | Locality |
|---|---|---|---|---|---|
| RCS | A63.934 | Old adult | Wild | *verus* | Sierra Leone |
| RCS | A63.935 | Old adult | Wild | *verus* | Sierra Leone |
| RCS | A63.932 | Prime adult | Wild | *verus* | Sierra Leone |
| RCS | A63.95 | Prime adult | Wild | *verus* | Sierra Leone |
| Smithsonian | USNM.450071 | Prime to old adult | Wild | *verus* | Cote d'Ivoire |
| Smithsonian | USNM.481803 | Prime to old adult | Wild | *verus* | Liberia |
| Smithsonian | USNM.477333 | Prime to old adult | Wild | *verus* | Cote d'Ivoire |
| Powell-Cotton | NH.MER33.273.1 | Prime to old adult | Wild | *troglodytes* | Cameroon |
| Powell-Cotton | NH.MER33.506.1 | Prime to old adult | Wild | *troglodytes* | Cameroon |
| Powell-Cotton | NH.MER33.677.1 | Prime to old adult | Wild | *troglodytes* | Cameroon |
| Powell-Cotton | NH.MER33.720.1 | Prime to old adult | Wild | *troglodytes* | Cameroon |
| Powell-Cotton | NH.MER33.742.1 | Prime to old adult | Wild | *troglodytes* | Cameroon |
| Powell-Cotton | NH.MER35.171.1 | Prime to old adult | Wild | *troglodytes* | Cameroon |
| Powell-Cotton | NH.MER35.78.1 | Prime to old adult | Wild | *troglodytes* | Cameroon |
| Powell-Cotton | NH.MER.234.1 | Prime to old adult | Wild | *ellioti* | Cameroon |
| Powell-Cotton | NH.MER.277.1 | Prime to old adult | Wild | *ellioti* | Cameroon |
| Powell-Cotton | NH.MER.279.1 | Prime to old adult | Wild | *ellioti* | Cameroon |
| NMS | Z.2019.92.1 | Old adult | Captive | *verus* | United Kingdom |
| NMS | Z.2019.10.2 | Old adult | Captive | *verus* | United Kingdom |
| NMS | Z.2018.129.1 | Old adult | Captive | *verus* | United Kingdom |
| NMS | Z.2017.112 | Old adult | Captive | *verus* | United Kingdom |
| NMS | Z.2018.116.1 | Old adult | Captive | unknown | United Kingdom |
| NMS | Z.2019.23.1 | Old adult | Captive | unknown | United Kingdom |
| NMS | Z.2009.120 | Prime adult | Captive | unknown | United Kingdom |
| NMS | Z.2022.26.2 | Old adult | Captive | unknown | United Kingdom |
| NMS | Z.2022.26 | Prime adult | Captive | unknown | United Kingdom |
| NMS | Z.2022.24 | Old adult | Captive | unknown | United Kingdom |
| NMS | Z.2004.96.1 | Prime adult | Captive | *verus* | United Kingdom |
| NMS | Z.2005.33.1 | Prime adult | Captive | unknown | United Kingdom |
| NMS | Z.2009.119 | Prime adult | Captive | unknown | United Kingdom |
| NMS | Z.2009.125 | Prime adult | Captive | unknown | United Kingdom |
| NMS | Z.2014.140.2 | Prime adult | Captive | hybrid | United Kingdom |
| NMS | Z.2014.141.1 | Prime adult | Captive | hybrid | United Kingdom |
| NMS | Z.2016.122.2 | Prime adult | Captive | hybrid | United Kingdom |
| NMS | Z.2018.115.1 | Prime adult | Captive | unknown | United Kingdom |
| NMS | Z.2019.26.1 | Prime adult | Captive | unknown | United Kingdom |
| NMS | Z.2023.11 | Prime adult | Captive | unknown | United Kingdom |

## 1.3 Isotopic and Trace Element Analysis

### Stable Isotope Analysis

Stable isotope analysis was carried out on Choppers' nails, teeth and bones. Prior to analysis, all nail, teeth and bone samples were visually inspected for any contaminants and weighed. The samples were then washed in a pre-prepared 10ml Chloroform/Methanol (2:1) solution, before rinsing them three times in Ultra High Quality water (UHQ, 18MW) to remove lipids and external contaminants. The clean samples were then dried in the oven overnight at 35°C. The dried nails were sectioned at the distal end and proximal end with 1mm sections and weighed into tin capsules. Each nail section (≥ 0.2 mg) was transferred into 8 x 5mm tin capsules for isotopic measurement. The nail samples were run on average in triplicate using a Sercon Elemental Analyzer coupled to a Europa Geo 20–20 isotope ratio mass spectrometer at the University of Reading, UK.

The analysis of enamel carbonate was conducted following the procedure outlined by [2]. The enamel was finely ground using an agate mortar and pestle. Potential diagenetic contaminants were eliminated by soaking the enamel powder in 0.1ml of 0.1 M acetic acid per 1 mg of enamel and agitating for 10 minutes. The acid was then removed through repeated rinsing in distilled water six times, with centrifugation for 2 min at 13,700 x g between rinses. Each Eppendorf tube was covered with small parafilm sheets, and a small hole was made using a sharp object to facilitate appropriate drying of the sample. The samples were then frozen for 24 hours and freeze-dried for 24 hours to eliminate all water. Approximately 0.6-0.8 mg of enamel was used for measurements. The $CO_2$ produced was analysed with a Thermo Delta V Advantage isotope ratio mass spectrometer coupled to a Thermo gas chromatography-based GasBench II system via a Conflo IV interface at the Chemical Analysis Facility (CAF) of the University of Reading, UK.

Collagen extraction from the bone samples followed the Longin (1971)[3] method modified by Collins et al., (1998)[4]. Each bone sample was cleaned using a scalpel to remove contaminants of the outer layer of bone. Following this, bone samples of about 250-450 mg were demineralised for 2-4 days in 0.5M hydrochloric acid (HCl), rinsed thrice using deionised water and then gelatinised using pH3 HCl on hot blocks at 70°C for 48 hours. Next, the supernatant liquid containing the collagen was filtered using 60-90μm Eezee® filters to remove unwanted particulate matter from the collagen solution, and was then frozen for a minimum of 12 hours at -20°C before being freeze-dried for 48 hours. Approximately 0.6-0.8mg of freeze-dried retentate was weighed out into 8x5 mm tin capsules and combusted alongside standards in a

Sercon Elemental Analyzer coupled to a Europa Geo 20–20 isotope ratio mass spectrometer at the University of Reading, UK.

Accuracy for isotope analysis was determined through a two-point calibration curve with drift correction, using internal working standards calibrated to internationally certified reference materials within each analytical run. These included the amino acid methionine (Elemental Microanalysis/MethR), powdered Bovine Liver Standard (NIST1577a-BLS) and a batch of pork gelatine and fish skin prepared at the Reading stable isotope laboratory (Reading pork gelatine—RPG; accepted values: $\delta^{15}N$ = 5.04‰, $\delta^{13}C$ = − 21.54‰; Reading fish skin—RFS; accepted values: $\delta^{15}N$ = 13.99‰, $\delta^{13}C$ = − 15.64‰). All $\delta^{15}N$ and $\delta^{13}C$ values are reported in per mil relative to the atmospheric nitrogen (AIR) and Vienna PeeDee Belemnite standard (VPDB) respectively.

Reconstructing Choppers' diet throughout her life using different tissues required the application of the isotopic offset between diet and tissue on $\delta^{13}C$ and $\delta^{15}N$ values (*Table S2*). All $\delta^{13}C$ values were Suess corrected using Long et al., (2005)[5]. The $\delta^{18}O$ values were converted to drinking water values using Chenery et al., (2012)[6] equation:

$$\delta^{18}O_{DW} = 1.590 \times \delta^{18}O_{C} - 48.634)$$

*Table S2: Stable carbon and nitrogen isotopic fractionation differences used for enamel, bone, hair and nails.*

| | **CARBON** | | | **NITROGEN** |
|---|---|---|---|---|
| | Enamel carbonate | Bone collagen | Hair and Nails | Bone collagen, Hair and Nails |
| | $\delta^{13}C_{enamel\ carbonate}$‰ relative to $\delta^{13}C_{diet}$‰ | $\delta^{13}C_{bone\ collagen}$‰ relative to $\delta^{13}C_{diet}$‰ | $\delta^{13}C_{hair/nail}$ ‰ relative to $\delta^{13}C_{diet}$‰ | $\delta^{15}N_{bone\ collagen}$‰ relative to $\delta^{15}N_{diet}$‰ |
| **ISOTOPIC OFFSET BETWEEN DIET AND TISSUE** | 11.5‰ | 5‰ | 3‰ | 3‰ |
| **REFERENCE** | [7] | [7] | [8] | [7,8] |

## Trace Element Analysis

Trace element analysis was carried out using a ThermoFisher Niton XL3 Portable XRF Analyser, recording a total of about 50 elements. Four elements, strontium (Sr), barium (Ba), zinc (Zn), and iron (Fe) were chosen to complement dietary reconstruction via stable isotopes during the first seven years of Choppers' life. Strontium (Sr) and barium (Ba) metabolism closely follow calcium (Ca) in the ecosystem and are routed from the whole diet to the skeletal tissues of an animal [9,10]. Both Sr/Ca and Ba/Ca decrease with each increasing trophic position and therefore, higher Sr/Ca and Ba/Ca ratios are used as indicators of vegetable intake [11]. On the other hand, lower Sr/Ca and Ba/Ca ratios are indicators of meat and marine-based diet [12–14]. The relative contractions of Sr to Ca (Sr/Ca) and Ba to Ca (Ba/Ca) have thus been utilised to reconstruct diet.

Trace elements were measured in parts per million (PPM) using a portable X-Ray fluorescence (pXRF) analyser and were converted to moles using the following equation:

Molarity=PPM/molar mass/1000

The calcium (Ca) concentration was used to normalise all the elements' values so as to provide standardised values for better comparing dietary habits.

# Extended Results

## 2.1 Photographs of Skeleton

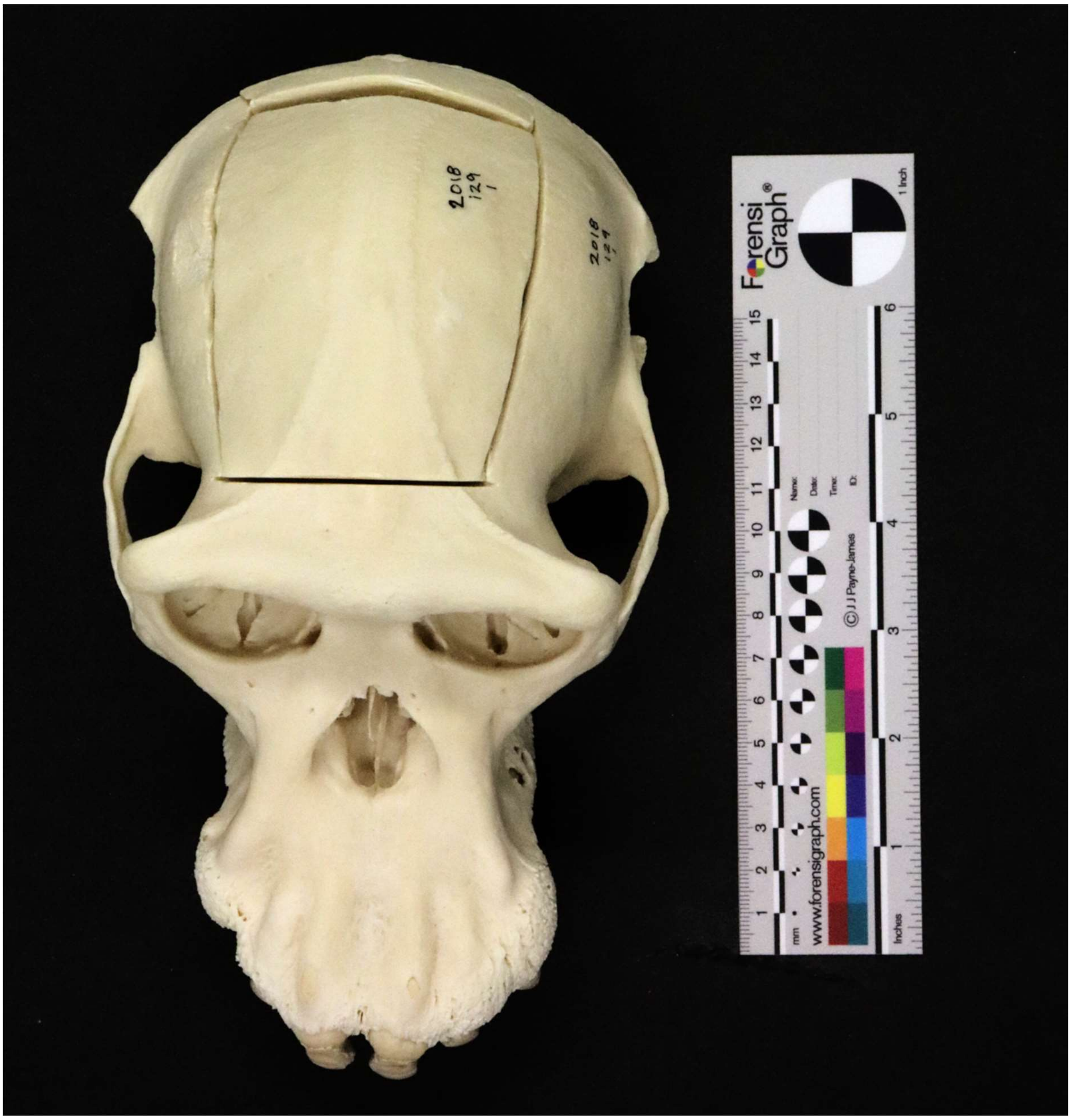


*Figure S3: Choppers' cranium, dorsal view.*

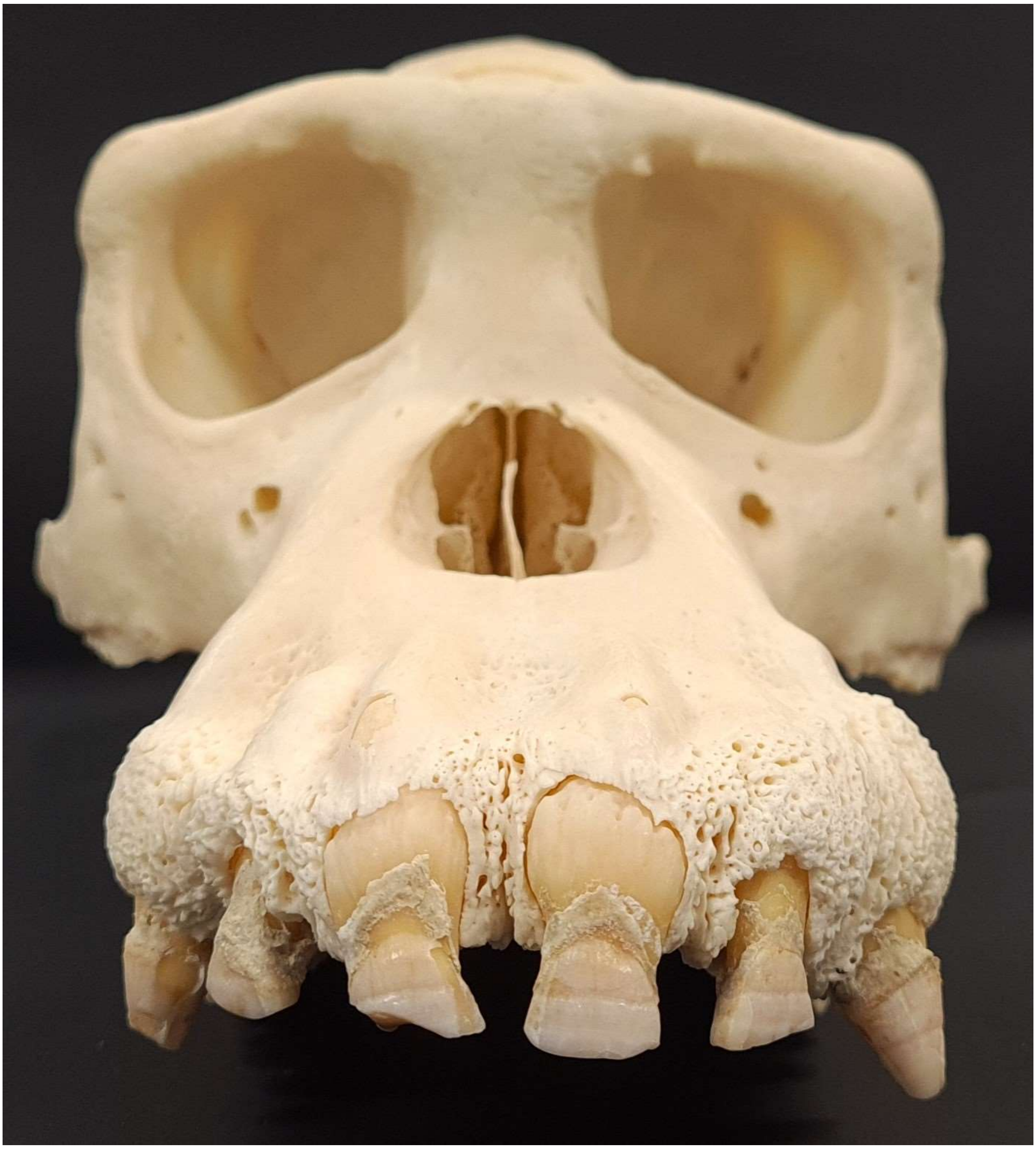

*Figure S4: Choppers' cranium, frontal view.*

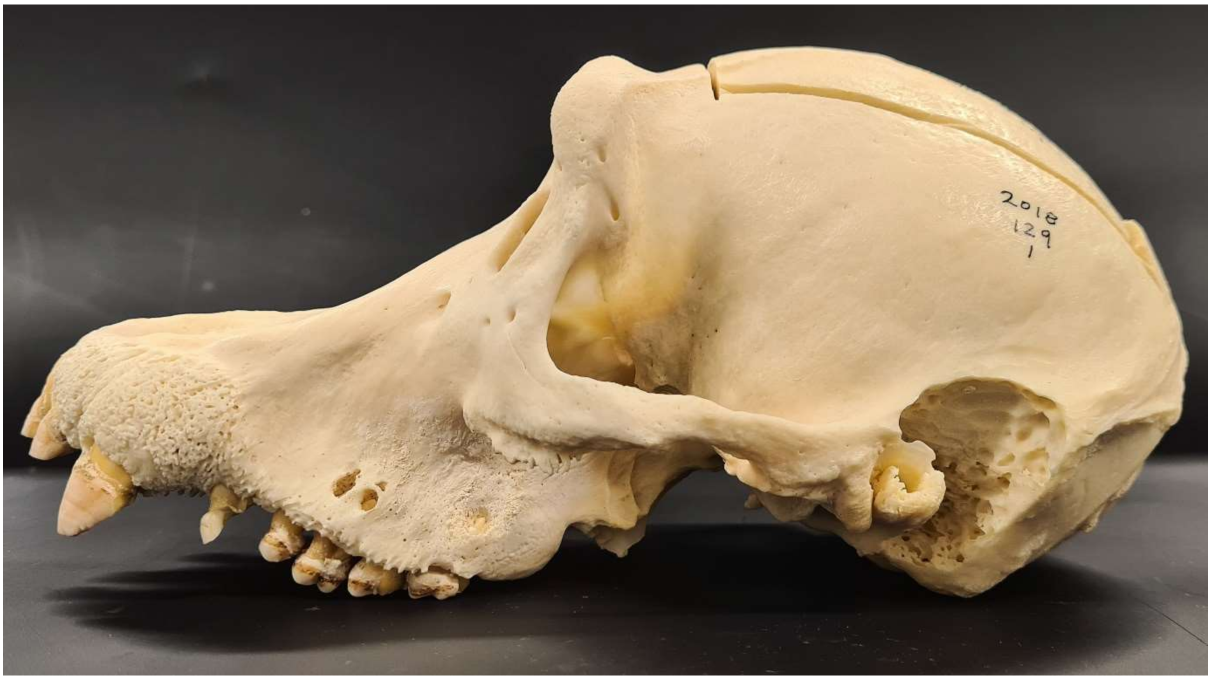


*Figure S5: Choppers' cranium, left lateral view.*

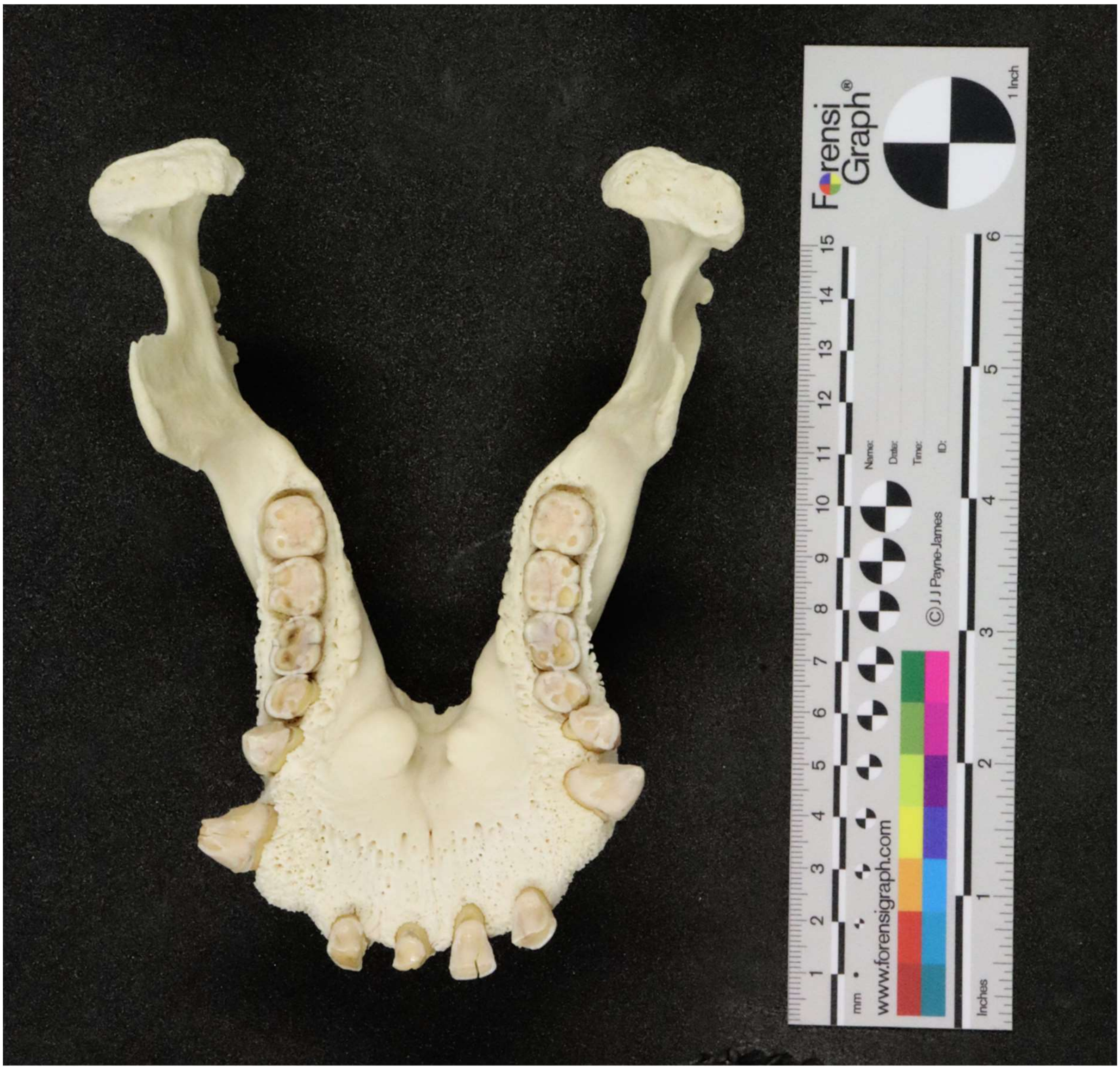

*Figure S6: Choppers' mandible, dorsal view.*

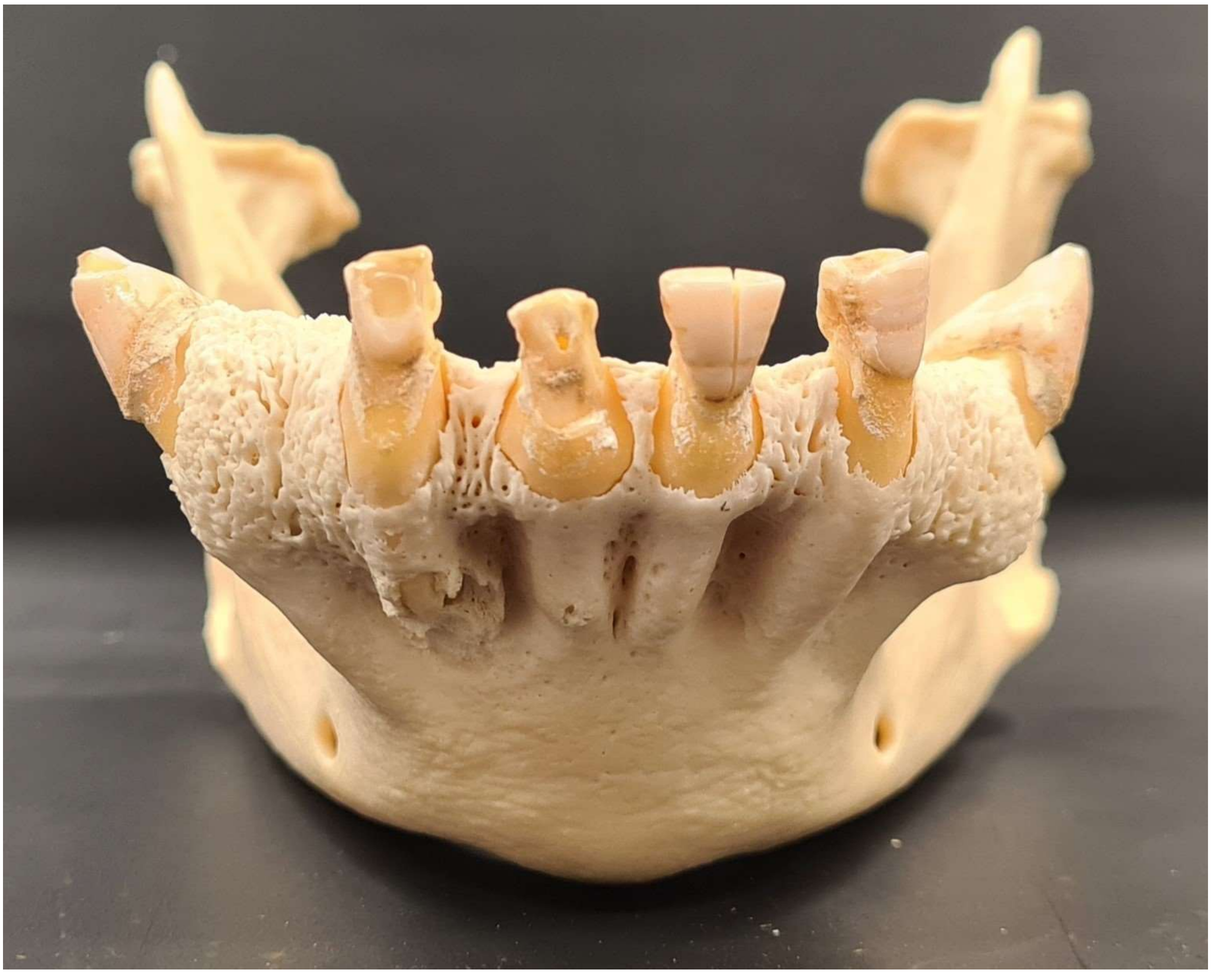

*Figure S7: Choppers' mandible, frontal view.*

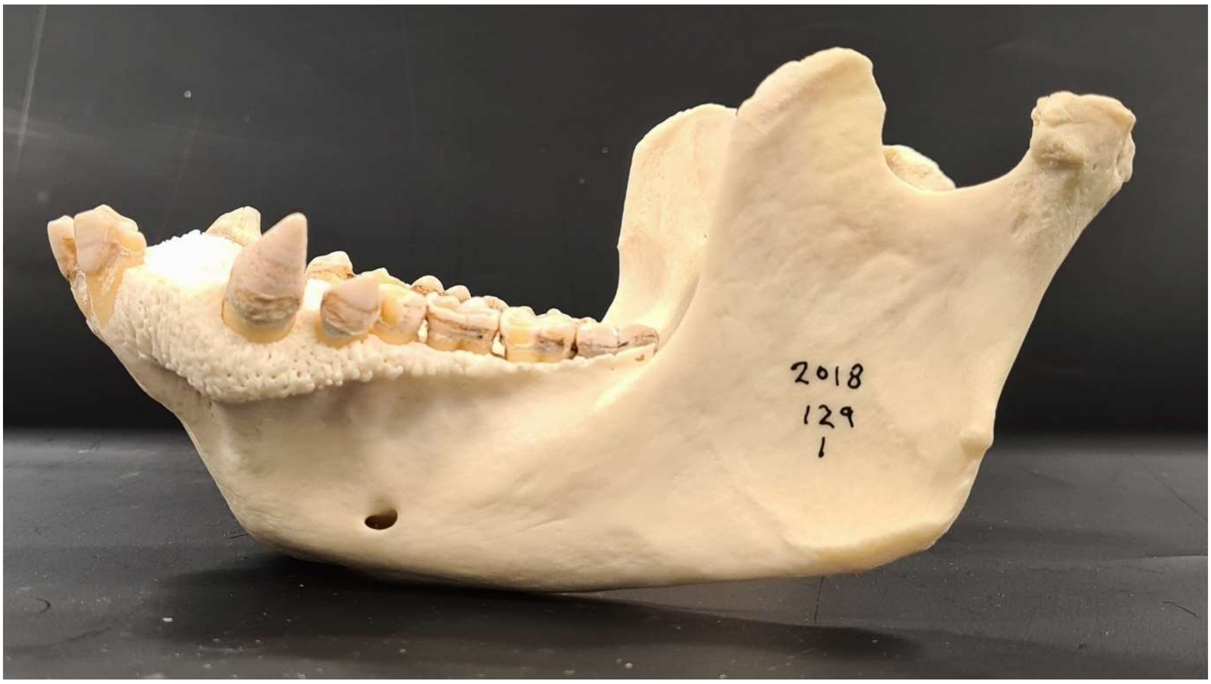


*Figure S8: Choppers' mandible, left lateral view.*

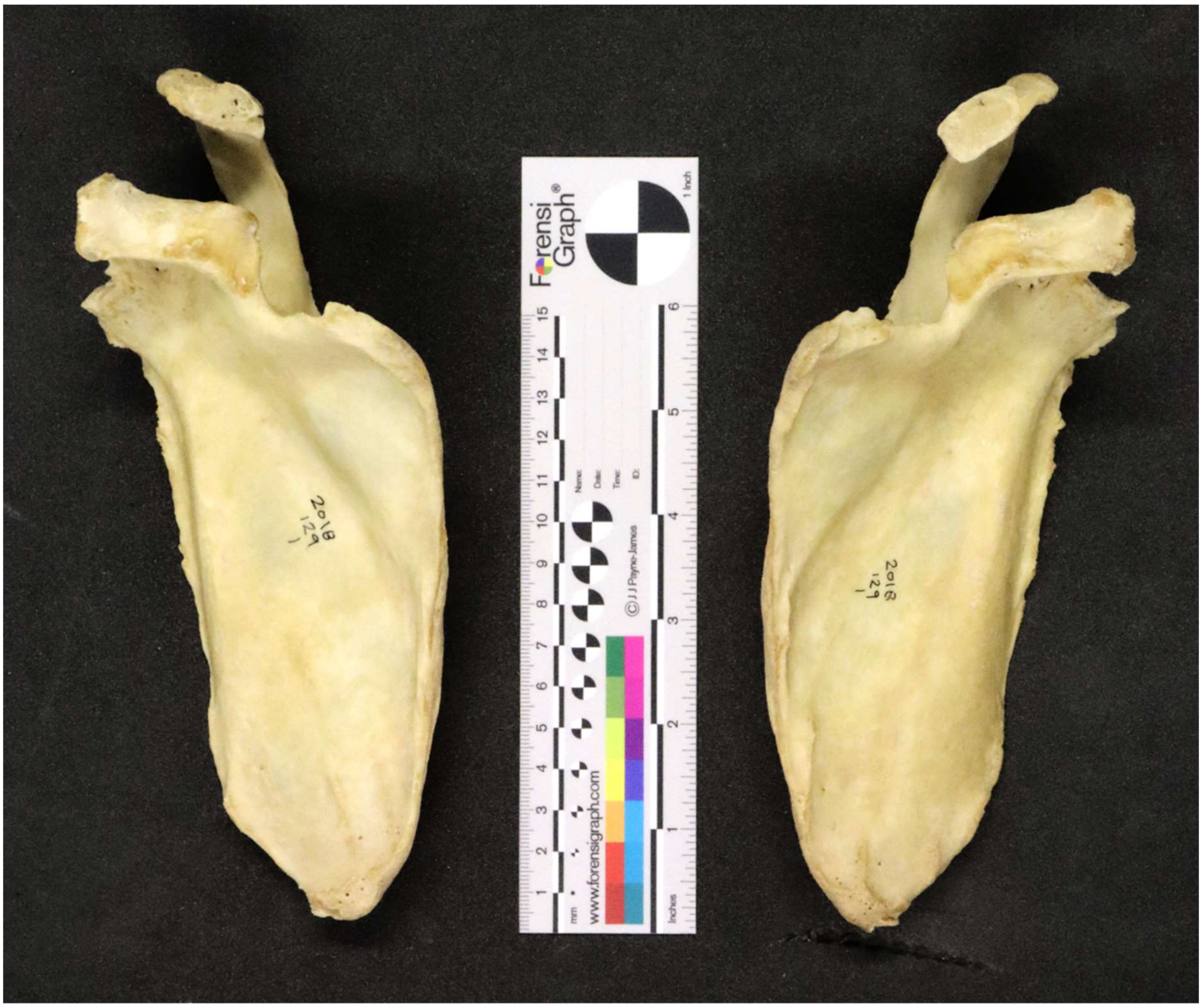

*Figure S9: Choppers' scapulae, ventral view.*

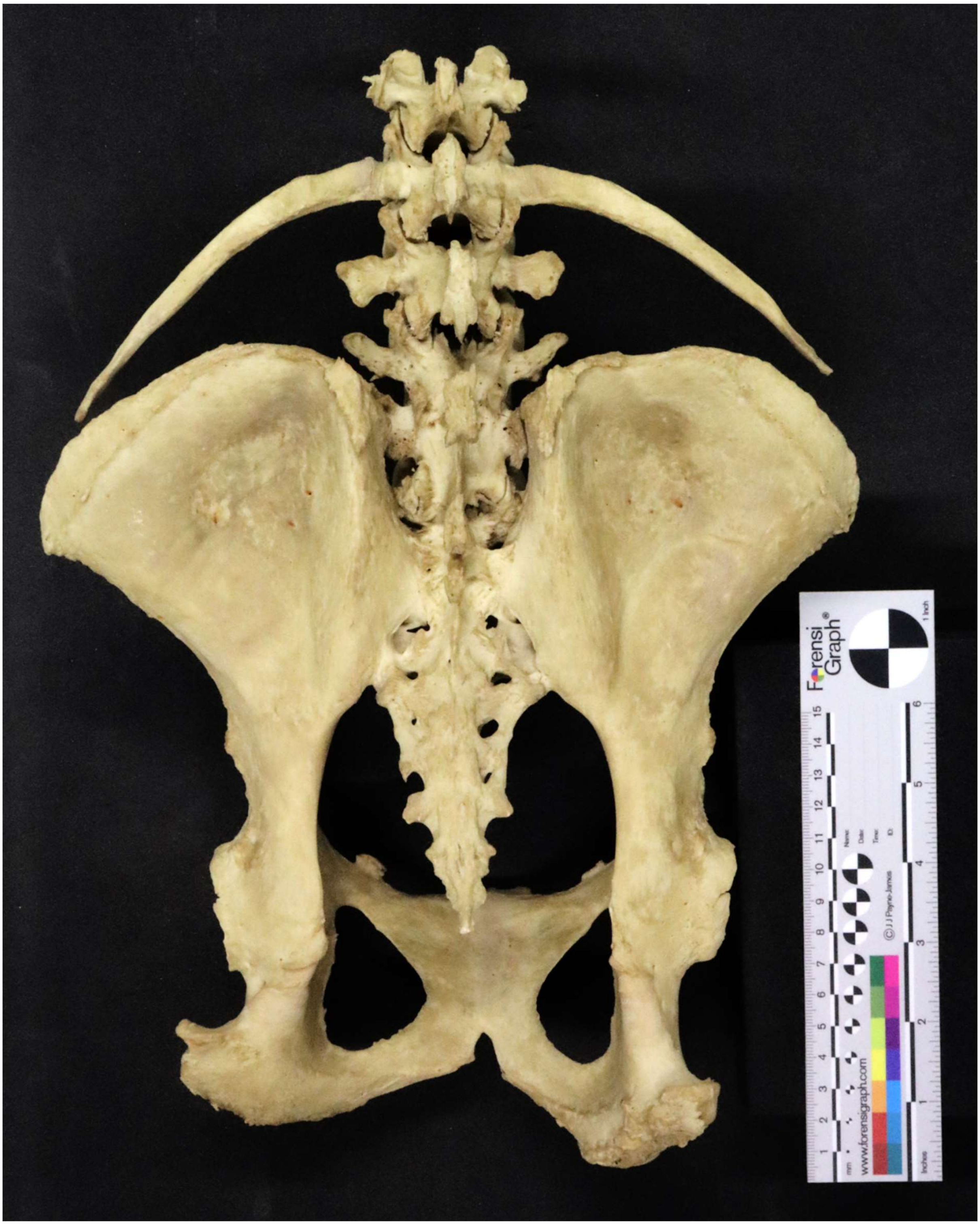


*Figure S10: Choppers' pelvis and fused vertebrae, ventral view.*

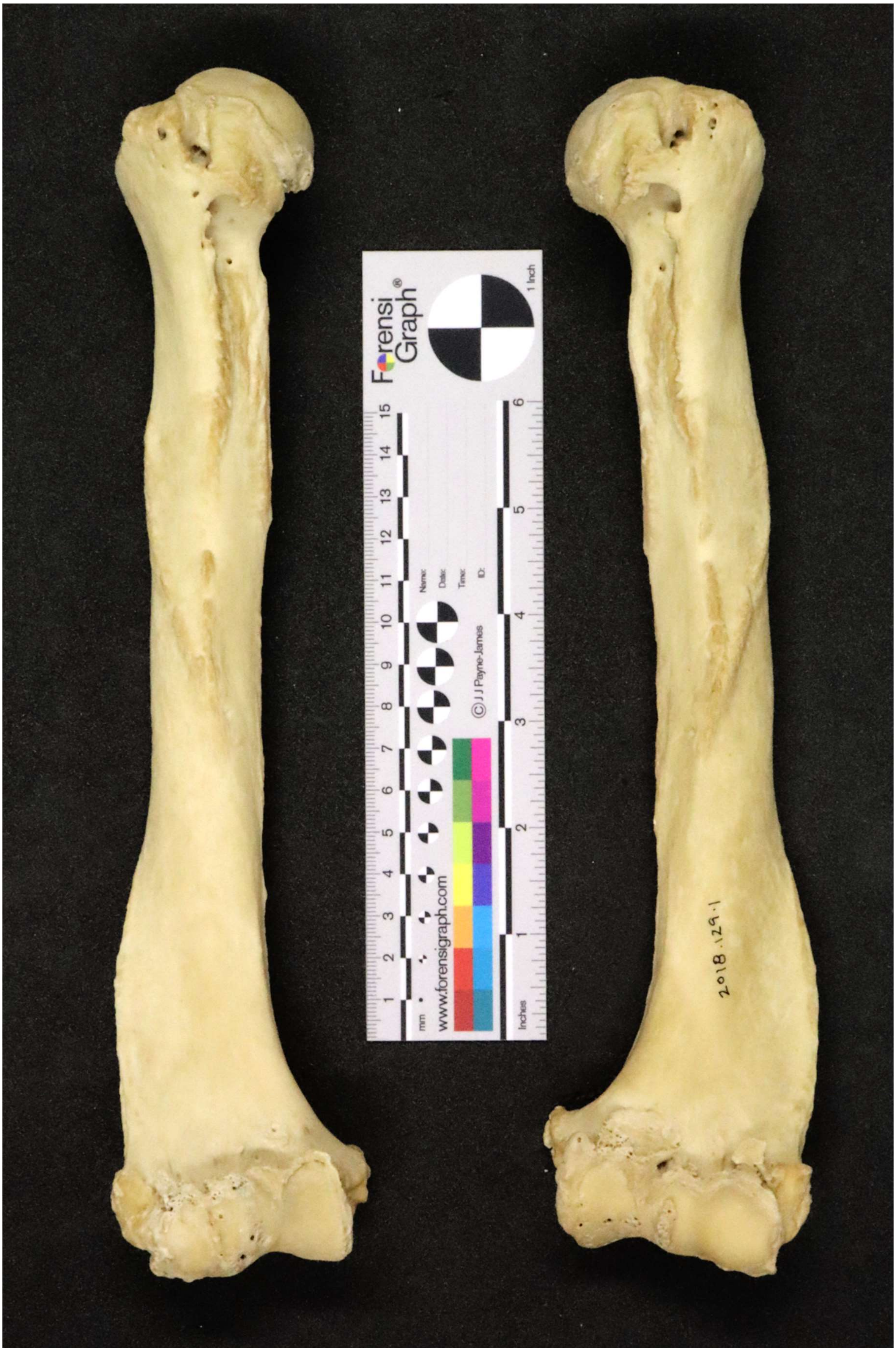

*Figure S11: Choppers' humeri, ventral view.*

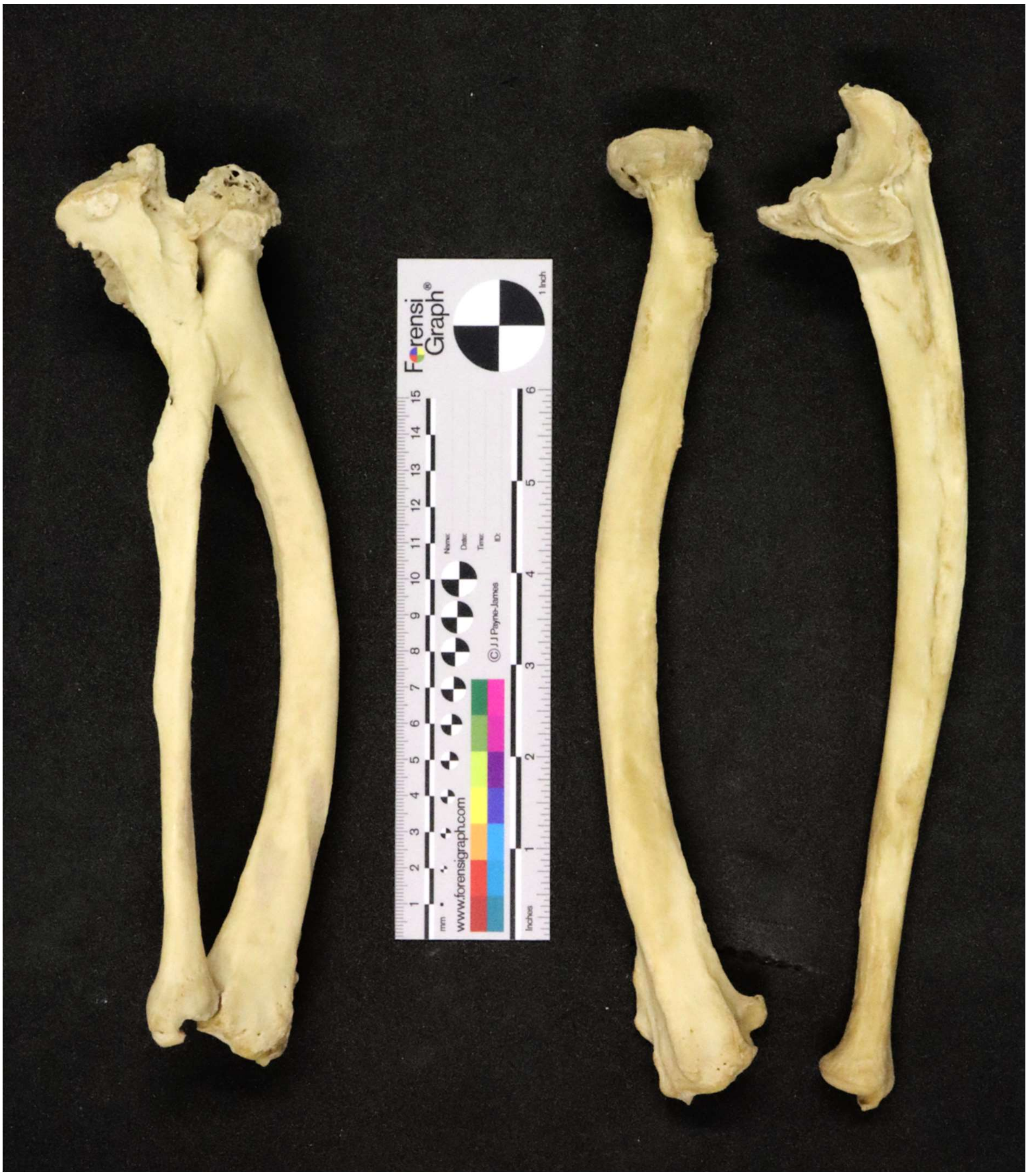


*Figure S12: Choppers' radii and ulnae, ventral view.*

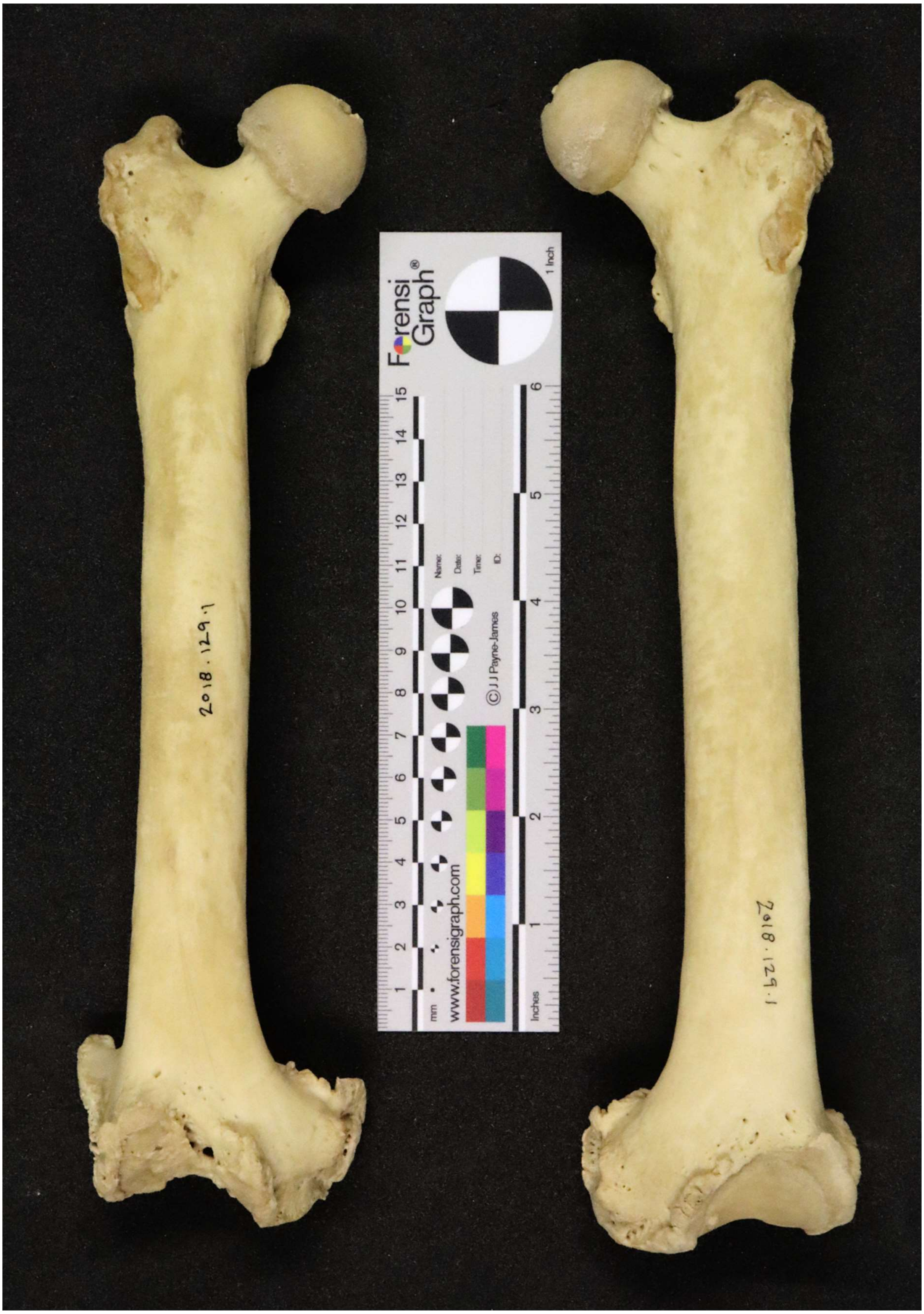

*Figure S13: Choppers' femora, ventral view.*

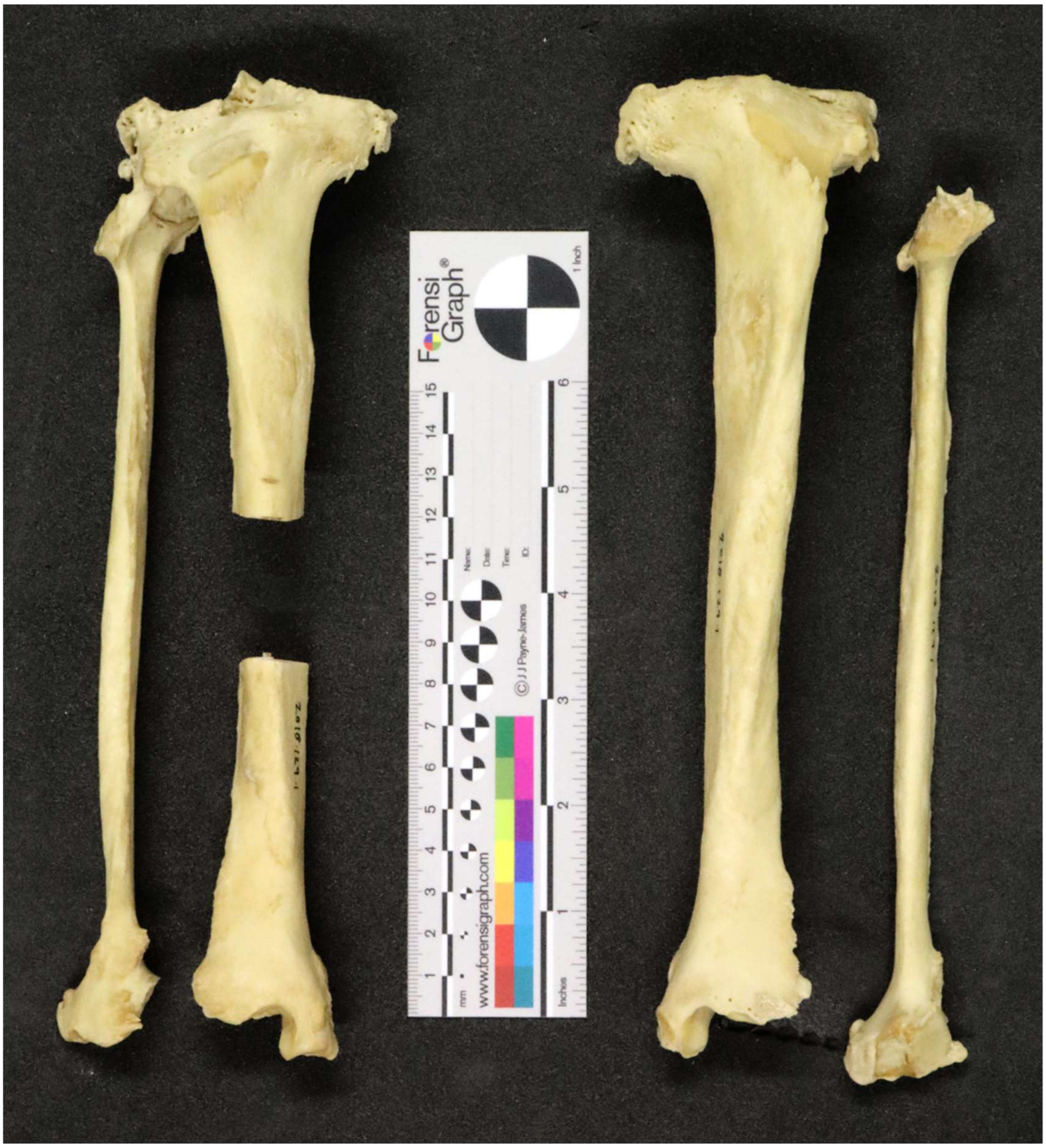


*Figure S14: Choppers' tibiae and fibulae, ventral view.*

## 2.2 Photographs in Life

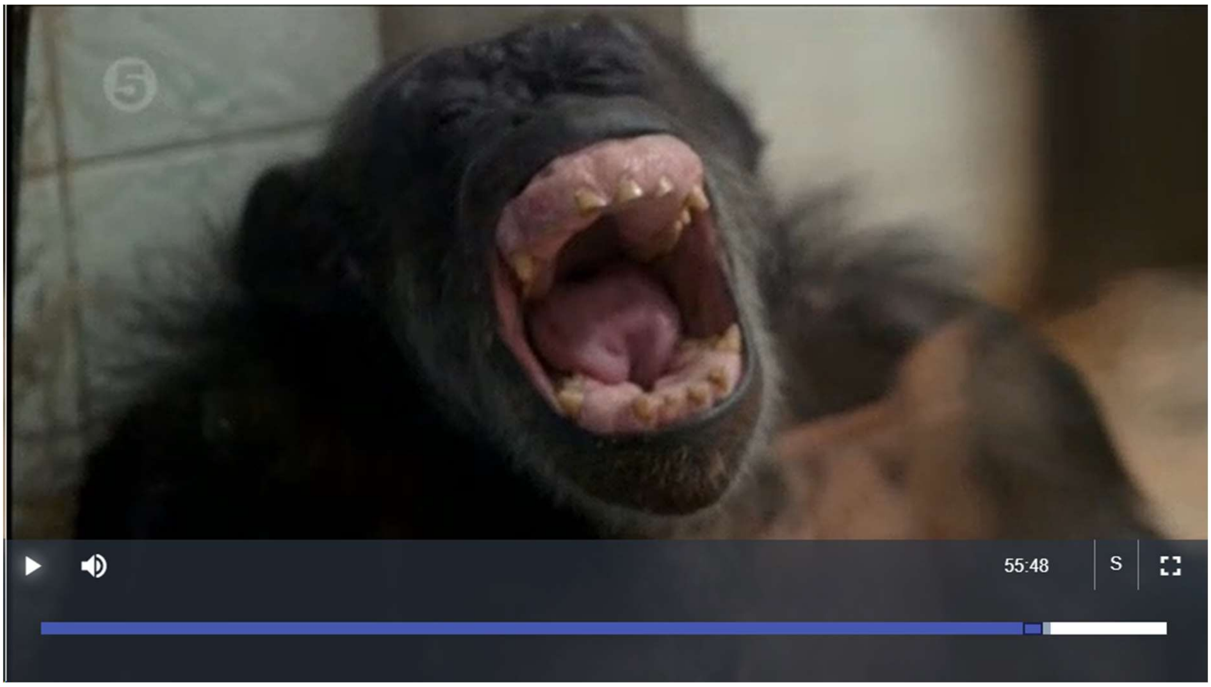


*Figure S15: Screenshot of Choppers yawning from Channel 5's 'Secrets of the Tea Chimps'* [15]*. This image shows inflammation of Choppers' gum which has covered her right lateral incisor.*

# Dentin Exposure Results

*Table S3: Results of dentin exposure analysis of the occlusal surface of Choppers' molars and premolars. Estimates of total occlusal surface and total exposed dentin were made where postmortem tooth damage had occurred in small discrete locations e.g. to the edge of the occlusal surface where enamel had been removed for isotopic analysis.*

| Structure | Side | Tooth | Exposed Dentin | Notes |
|---|---|---|---|---|
| Maxilla | Left | P3 | NA | Postmortem breakage |
| Maxilla | Left | P4 | NA | Postmortem breakage |
| Maxilla | Left | M1 | 25% | Light postmortem breakage, allowing for exposed dentin estimation |
| Maxilla | Left | M2 | 33% | Light postmortem breakage, allowing for exposed dentin estimation |
| Maxilla | Left | M3 | NA | Postmortem breakage |
| Maxilla | Right | P3 | 29% | Good preservation condition |
| Maxilla | Right | P4 | 35% | Good preservation condition |
| Maxilla | Right | M1 | 25% | Good preservation condition |
| Maxilla | Right | M2 | 22% | Good preservation condition |
| Maxilla | Right | M3 | 12% | Light postmortem breakage, allowing for exposed dentin estimation |
| Mandible | Left | P3 | 1% | Good preservation condition |
| Mandible | Left | P4 | 16% | Light postmortem breakage, allowing for exposed dentin estimation |
| Mandible | Left | M1 | 27% | Light postmortem breakage, allowing for exposed dentin estimation |
| Mandible | Left | M2 | 13% | Light postmortem breakage, allowing for exposed dentin estimation |
| Mandible | Left | M3 | 6% | Good preservation condition |
| Mandible | Right | P3 | 0 | Good preservation condition, no exposed dentin |
| Mandible | Right | P4 | NA | Postmortem breakage |
| Mandible | Right | M1 | 41% | Light postmortem breakage, allowing for exposed dentin estimation |
| Mandible | Right | M2 | 22% | Light postmortem breakage, allowing for exposed dentin estimation |
| Mandible | Right | M3 | 5% | Good preservation condition |

## 2.3 Pathology Score Tables

*Table S4: Pathology scores of the spinal column, following Law and Kitchener (2020) [16]. Scores are presented for both Spondyloarthroses (SA) and for Diffuse Idiopathic Skeletal Hyperostosis (DISH). Scores range from 0 (no osteophytes) to five (fusion of joints by osteophytes).*

| | Anterior zygapophyses - SA | Posterior zygapophyses - SA | Anterior centrum - DISH | Posterior centrum - DISH | Rib articulation - SA |
|---|---|---|---|---|---|
| **Cervical 1** | 1 | 1 | 1 | 0 | |
| **Cervical 2** | 1 | 2 | 1 | 3 | |
| **Cervical 3** | 1 | 2 | 0 | 3 | |
| **Cervical 4** | 1 | 1 | 2.5 | 3.5 | |
| **Cervical 5** | 1 | 1 | 3 | 3 | |
| **Cervical 6** | 1 | 1 | 2.5 | 1 | |
| **Cervical 7** | 1 | 1 | 2 | 1 | |
| **Thoracic 1** | 1 | 0 | 1.5 | 1 | 2.5 |
| **Thoracic 2** | 0 | 0 | 1 | 2 | 2 |
| **Thoracic 3** | 0 | 1 | 1.5 | 0 | 2 |
| **Thoracic 4** | 1 | 1 | 2.5 | 3 | 2.5 |
| **Thoracic 5** | 1 | 1 | 2 | 2.5 | 2 |
| **Thoracic 6** | 1 | 1 | 2 | 3 | 1.5 |
| **Thoracic 7** | 1 | 1 | 2.5 | 3.5 | 1.5 |
| **Thoracic 8** | 0 | 0 | 3 | 4 | 1.5 |
| **Thoracic 9** | 1 | 1 | 4 | 4 | 1.5 |
| **Thoracic 10** | 1 | 1 | 4 | 3.5 | 2 |
| **Thoracic 11** | 1.5 | 1 | 4 | 2 | 0 |
| **Thoracic 12** | 2 | 2 | 2.5 | 1.5 | 2.5 |
| **Lumbar 1** | 1 | 1.5 | 2.5 | 5 | 3 |
| **Lumbar 2** | 1 | 2 | 5 | 5 | 5 |
| **Lumbar 3** | 1 | 1.5 | 5 | 5 | |
| **Lumbar 4** | 2.5 | 5 | 5 | 5 | |
| **Lumbar 5** | 5 | 3 | 5 | 5 | |
| **Sacrum** | 2 | n/a | 5 | n/a | |

*Table S5: Arthroses pathology scores of the postcranial skeleton, (excluding the spinal column), following Law and Kitchener (2020) [16]. Scores range from 0 (no osteophytes) to five (fusion of joints by osteophytes).*

| | Proximal | Distal | Other |
|---|---|---|---|
| **Humerus L** | 2 | 3.5 | |
| **Humerus R** | 2.5 | 2.5 | |
| **Radius L** | 1 | 2 | |
| **Radius R** | 4.5 | 1.5 | distorted |
| **Ulna L** | 4 | 1 | |
| **Ulna R** | 3.5 | 2 | very reduced and distorted |
| **Femur L** | 1 | 2.5 | |
| **Femur R** | 0 | 4.5 | |
| **Tibia L** | 2.5 | 2.5 | |
| **Tibia R** | 3.5 | 1 | |
| **Fibula L** | 2.5 | 2.5 | |
| **Fibula R** | 4.5 | 1.5 | |
| **Scapula L** | 3 | n/a | |
| **Scapula R** | 3.5 | n/a | |
| **Clavicle L** | 0 | 2 | |
| **Clavicle R** | 0 | 2 | |
| **Patellae** | 4 | | |

## 2.4 Geometric Morphometrics Results

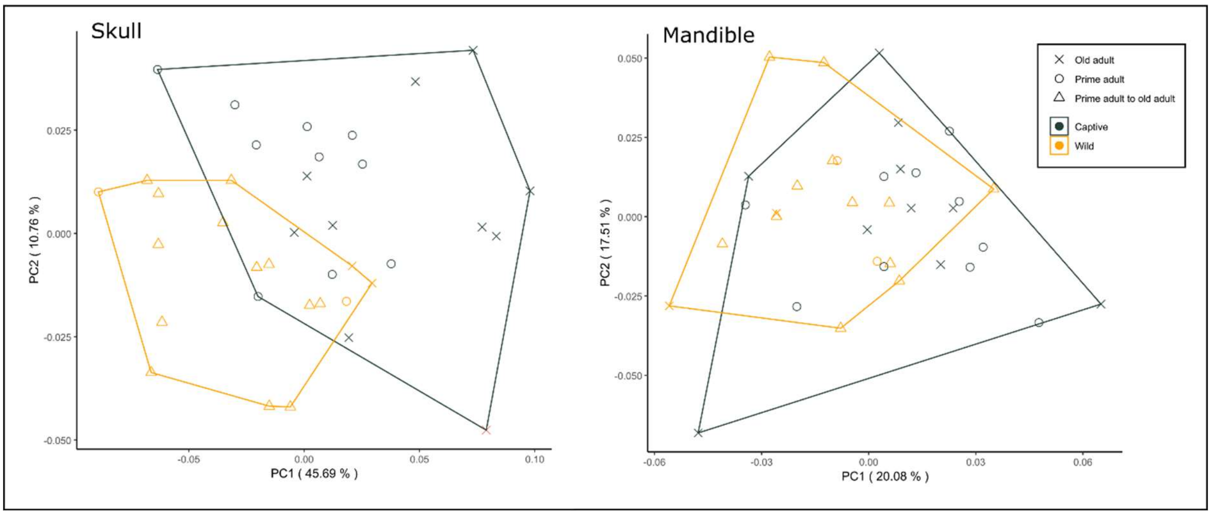


*Figure S16: The results of geometric morphometric analyses of the skull and mandible of 37 captive and wild female chimpanzees. Here, we present the results (see Figure 3) by age class of the specimen, categorised as Prime adult (13-30 years old) and Old adult (30+ years old).*

## 2.5 Linear Morphometrics Results

*Table S6: Linear long bone measurements and live maximum body weight (Species 360: Zoological Information Management System)[17] of captive chimpanzee specimens housed at the National Museums Scotland.*

| Register no. | Sex | Age class | Origin | Maximum Weight (kg) | Right Femur Length (mm) | Left Femur Length (mm) | Left Humerus Length (mm) | Right Humerus Length (mm) |
|---|---|---|---|---|---|---|---|---|
| NMS.Z.2019.23.1 | F | Old adult | Monkey World | | 309.56 | 307.73 | 292.03 | 293.6 |
| NMS.Z.2017.112 | F | Old adult | Chester | | 309.1 | 307.05 | 306.56 | 310.42 |
| NMS.Z.2014.141.1 | F | Prime adult | Chester | 55.5 | 303.3 | 299.26 | 293.36 | 290.04 |
| NMS.Z.2023.11 | F | Prime adult | Colchester Zoo | 47.7 | 302.29 | 298.39 | 304.63 | 295.36 |
| NMS.Z.2003.102 | F | Old adult | London Zoo | 50 | 299.77 | 296.5 | 295.3 | 294.36 |
| NMS.Z.2022.26 | F | Old adult | Monkey World | 44 | 298.86 | 287.4 | 291.18 | 290.84 |
| NMS.Z.2019.10.2 | F | Old adult | Edinburgh Zoo | 68 | 298.7 | 299.51 | 294.5 | 298.91 |
| NMS.Z.2019.23.2 | F | Adolescent | Monkey World | 59 | 296.75 | 299.84 | 298.67 | 298.81 |
| NMS.Z.2003.79.2 | F | Adolescent | Twycross Zoo | | 294.44 | 291.43 | 278.1 | 279.96 |
| NMS.Z.2005.33.1 | F | Prime adult | Monkey World | | 293.29 | 293.45 | 281.65 | 285.32 |
| NMS.Z.2019.92.1 | F | Old adult | Twycross Zoo | 48 | 292.59 | 290.54 | 307.04 | 306.39 |
| NMS.Z.2018.115.1 | F | Prime adult | Monkey World | | 291.64 | 294.2 | 301.08 | 303.11 |
| NMS.Z.2014.140.1 | F | Old adult | Twycross Zoo | 50 | 291.5 | 289.28 | 288.39 | 288.65 |
| NMS.Z.2022.24 | F | Old adult | Colchester Zoo | 48 | 290.27 | 293.45 | 294.64 | 295.21 |
| NMS.Z.2004.209 | F | Old adult | London Zoo | | 288.5 | 285.31 | 294.77 | 294.99 |
| NMS.Z.2022.26.2 | F | Old adult | Monkey World | 65 | 287.78 | 289.59 | 282.96 | 287.91 |
| NMS.Z.2016.122.1 | F | Old adult | Twycross Zoo | 73.5 | 284.26 | 287.05 | 286.13 | 293.07 |

| | | | | | | | | |
|---|---|---|---|---|---|---|---|---|
| NMS.Z.2019.26.1 | F | Prime adult | Monkey World | 54.1 | 284.05 | 287.44 | 280.49 | 284.27 |
| NMS.Z.2009.125 | F | Prime adult | Belfast | 56 | 280.37 | 281.07 | 281.71 | 279.34 |
| NMS.Z.2016.122.2 | F | Prime adult | Twycross Zoo | 60 | 279.64 | 280.65 | 292.64 | 291.21 |
| NMS.Z.2004.96.1 | F | Prime adult | Edinburgh Zoo | | 274.23 | 275.35 | 279.15 | 274.75 |
| NMS.Z.2009.119 | F | Prime adult | Twycross Zoo | | 271.19 | 269.96 | 255.74 | 260.16 |
| NMS.Z.2022.28.1 | F | Old adult | Twycross Zoo | 82 | 263.33 | 261.7 | 253.5 | 250.21 |
| NMS.Z.2014.140.2 | F | Prime adult | Twycross Zoo | 47.8 | 261.9 | 261.57 | 270.64 | 263.35 |
| NMS.Z.2009.120 | F | Old adult | Belfast | | 261.01 | 262.96 | 269.65 | 267.98 |
| NMS.Z.2018.129.1 | F | Old adult | Twycross Zoo | 55 | 260.3 | 271.32 | 275.93 | 279.75 |
| NMS.Z.2018.116.1 | F | Old adult | Chester | 22.5 | 252.44 | 251.9 | 255.64 | 253.38 |
| NMS.Z.2022.26.4 | F | Adult | Monkey World | | 315.52 | 311.54 | 304.08 | 305.14 |
| NMS.Z.2022.25 | M | Prime adult | Belfast | | 390.19 | 391.89 | 374.94 | 383.44 |
| NMS.Z.2012.57.3 | M | Prime adult | Edinburgh Zoo | 70.6 | 328.76 | 327.07 | 337 | 335.7 |
| NMS.Z.2004.96.2 | M | Prime adult | Edinburgh Zoo | 70.2 | 324.52 | 325.92 | 319.44 | 324.59 |
| NMS.Z.2018.115.4 | M | Prime adult | Monkey World | 60 | 321.46 | 322.86 | 314.48 | 310.01 |
| NMS.Z.2015.132 | M | Old adult | Africa Alive! Kessingland | | 321.02 | 319.19 | 312.82 | 316.04 |
| NMS.Z.2018.115.3 | M | Old adult | Monkey World | 70 | 319.33 | 323.93 | 315.3 | 317.51 |
| NMS.Z.2012.36.1 | M | Prime adult | Welsh Mountain Zoo | | 318.25 | 311.09 | 312.98 | 312.21 |
| NMS.Z.2012.57.2 | M | Prime adult | Edinburgh Zoo | 57.8 | 317.57 | 318.35 | 319.07 | 323.91 |
| NMS.Z.2012.60 | M | Old adult | Welsh Mountain Zoo | 80 | 315.82 | 311.66 | 308.37 | 309.31 |
| NMS.Z.2008.123 | M | Prime adult | Warsaw Zoo | 50.8 | 311.97 | 311.67 | 303.17 | 304.12 |
| NMS.Z.2012.57.1 | M | Old adult | Edinburgh Zoo | 55.5 | 309.17 | 305 | 311.51 | 313.24 |
| NMS.Z.2014.142 | M | Adolescent | London Zoo | 50.2 | 308.13 | 309.47 | 307.84 | 308.19 |
| NMS.Z.2019.92.2 | M | Old adult | Twycross Zoo | 75 | 305.69 | 304.82 | 303.61 | 304.99 |
| NMS.Z.2006.46.1 | M | Prime adult | Whipsnade Zoo | | 299.78 | 298.15 | 323.34 | 322.51 |
| NMS.Z.2009.121 | M | Prime adult | Whipsnade Zoo | 60.8 | 296.86 | 300.1 | 305.68 | 308.9 |
| NMS.Z.1981.73 | M | Old adult | Edinburgh Zoo | | 295.23 | 297.64 | 290.7 | 295.45 |
| NMS.Z.2019.92.3 | M | Old adult | Twycross Zoo | 61 | 294.86 | 298.69 | 302.62 | 303.67 |
| NMS.Z.2022.26.3 | M | Adult | Monkey World | | 288.51 | 294.16 | 291.08 | 291.26 |
| NMS.Z.2022.27 | M | Old adult | Whipsnade Zoo | 74 | 287.67 | 276.3 | N/A | 282.56 |
| NMS.Z.2018.129.2 | M | Prime adult | Twycross Zoo | 63.5 | 286.9 | 286.95 | 281.95 | 280.56 |
| NMS.Z.2008.38 | M | Old adult | Edinburgh Zoo | | 285.61 | 286.28 | 285.59 | 288.7 |
| NMS.Z.2015.133 | M | Old adult | Flamingo Land | | 284.67 | 285.1 | 284.38 | 278.24 |
| NMS.Z.2005.107 | M | Prime adult | Monkey World | | 280.33 | 278.75 | 258.63 | 265.51 |
| NMS.Z.2018.115.2 | M | Prime adult | Monkey World | 54 | 277.44 | 272.93 | 275.6 | 280.65 |
| NMS.Z.2009.122 | M | Old adult | Monkey World | 37.5 | N/A | 303.28 | 290.68 | 292.29 |

## 2.6 Isotope and Trace Element Analysis Data

*Table S7: Isotope and trace element values for sample tissues. The age represented (yrs) is given as the midpoint of the emergence period.*

| Sample details | Age (yrs) | Trace element mol/mol ratios | | | | Isotopic Values (corrected) | | | |
|---|---|---|---|---|---|---|---|---|---|
| | | Sr/Ca | Ba/Ca | Zn/Ca | Fe/Ca | $\delta^{13}C_{diet}$ | $\delta^{15}N_{diet}$ | $\delta^{18}O_{dw}$ | $\delta^{34}S$ |
| **1st Molar (0-2yrs b.)** | 0.825 | 0.00062 | 0.00133 | 0.00063 | 0.00065 | -25.92 | | -9.9 | |
| **Incisors (0.5-3.5 yrs b.)** | 2 | 0.000518 | 0.0007878 | 0.0010927 | 0.000537 | | | | |
| **2nd Molar (1.5-4 yrs b.)** | 3.195 | 0.00076 | 0.00135 | 0.00108 | 0.00081 | | | | |
| **2nd Pre-molar (3.5-4 yrs b.)** | 3.13 | 0.00124 | 0.00227 | 0.00134 | 0.0012 | -25.00 | | -5.04 | |
| **Canine (1.25-6/7 yrs b.)** | 3.47 | 0.0003358 | 0.000527 | 0.0011124 | 0.000548 | | | | |
| **3rd Molar (4-7 yrs b.)** | 5.18 | 0.00048 | 0.00079 | 0.0004 | 0.00051 | -26.70 | | -4.51 | |
| **Bone outer (5-10 yrs d.)** | 36.3 | 0.000112 | 0.0001507 | 0.0003322 | 0.003321 | -24.01 | 5.52 | | 5.88 |
| **Femur middle (5-10 yrs d.)** | 41.3 | | | | | -24.29 | 5.58 | | 6.04 |
| **Femur inner (5-10 yrs d.)** | 45.3 | | | | | -23.76 | 6.52 | | 5.79 |
| **Femur powder (5-10 yrs d.)** | 45.6 | | | | | -23.68 | 6.72 | | 4.78 |
| **Nail section 1.1** | 46.028 | | | | | -22.87 | 4.48 | | |
| **Nail section 1.2** | 46.028 | | | | | -23.17 | 3.84 | | |
| **Nail section 1.3** | 46.028 | | | | | -23.14 | 3.92 | | |
| **Nail section 1.4** | 46.028 | | | | | -22.08 | 3.22 | | |
| **Nail section 1.5** | 46.028 | | | | | -22.08 | 3.82 | | |
| **Nail section 2.1** | 46.056 | | | | | -23.21 | 4.91 | | |
| **Nail section 2.2** | 46.056 | | | | | -23.27 | 4.58 | | |
| **Nail section 3.1** | 46.086 | | | | | -22.61 | 4.10 | | |
| **Nail section 3.2** | 46.086 | | | | | -22.57 | 4.65 | | |
| **Nail section 3.3** | 46.086 | | | | | -22.20 | 3.68 | | |
| **Nail section 3.4** | 46.086 | | | | | -22.23 | 2.99 | | |
| **Nail section 4.1** | 46.111 | | | | | -22.66 | 4.25 | | |
| **Nail section 4.2** | 46.111 | | | | | -22.71 | 4.09 | | |
| **Nail section 4.3** | 46.111 | | | | | -23.23 | 3.95 | | |
| **Nail section 4.4** | 46.111 | | | | | -23.18 | 4.70 | | |
| **Nail section 4.5** | 46.111 | | | | | -22.94 | 4.56 | | |
| **Nail section 5.1** | 46.138 | | | | | -23.45 | 4.83 | | |
| **Nail section 5.2** | 46.138 | | | | | -23.27 | 4.53 | | |
| **Nail section 5.3** | 46.138 | | | | | -23.56 | 4.65 | | |
| **Nail section 5.4** | 46.138 | | | | | -23.50 | 4.53 | | |
| **Nail section 5.5** | 46.138 | | | | | -23.27 | 4.74 | | |
| **Nail section 6.1** | 46.168 | | | | | -22.94 | 4.34 | | |
| **Nail section 6.2** | 46.168 | | | | | -23.02 | 4.10 | | |
| **Nail section 6.3** | 46.168 | | | | | -23.22 | 4.09 | | |
| **Nail section 6.4** | 46.168 | | | | | -23.00 | 4.10 | | |

| | | | | | | | | |
|---|---|---|---|---|---|---|---|---|
| **Nail section 6.5** | 46.168 | | | | | -23.12 | 4.12 | |
| **Nail section 7.1** | 46.195 | | | | | -22.81 | 3.31 | |
| **Nail section 7.2** | 46.195 | | | | | -22.75 | 3.80 | |
| **Nail section 7.3** | 46.195 | | | | | -22.71 | 4.17 | |
| **Nail section 7.4** | 46.195 | | | | | -22.41 | 3.16 | |
| **Nail section 7.5** | 46.195 | | | | | -22.72 | 3.06 | |
| **Nail section 8.1** | 46.223 | | | | | -22.91 | 4.33 | |
| **Nail section 8.2** | 46.223 | | | | | -23.12 | 4.94 | |
| **Nail section 8.3** | 46.223 | | | | | -22.64 | 4.02 | |
| **Nail section 8.4** | 46.223 | | | | | -22.93 | 4.10 | |
| **Nail section 8.5** | 46.223 | | | | | -22.41 | 3.93 | |
| **Nail section 9.1** | 46.25 | | | | | -23.45 | 4.81 | |
| **Nail section 9.2** | 46.25 | | | | | -22.88 | 4.66 | |
| **Nail section 9.3** | 46.25 | | | | | -22.89 | 5.30 | |
| **Nail section 9.4** | 46.25 | | | | | -22.91 | 4.83 | |
| **Nail section 9.5** | 46.25 | | | | | -22.73 | 4.18 | |
| **Nail section 10.1** | 46.275 | | | | | -23.02 | 4.76 | |
| **Nail section 10.2** | 46.275 | | | | | -23.22 | 5.41 | |
| **Nail section 10.3** | 46.275 | | | | | -23.03 | 5.08 | |
| **Nail section 10.4** | 46.275 | | | | | -22.88 | 4.82 | |
| **Nail section 11.1** | 46.302 | | | | | -23.37 | 3.10 | |
| **Nail section 11.2** | 46.302 | | | | | -23.29 | 4.28 | |
| **Nail section 11.3** | 46.302 | | | | | -23.17 | 3.58 | |
| **Nail section 11.4** | 46.302 | | | | | -22.79 | 3.10 | |
| **Nail section 11.5** | 46.302 | | | | | -22.90 | 3.08 | |
| **Hair section 1** | 46.198 | | | | | -22.69 | 3.28 | |
| **Hair section 2** | 46.209 | | | | | -22.58 | 3.38 | |
| **Hair section 3** | 46.22 | | | | | -22.05 | 3.22 | |
| **Hair section 4** | 46.231 | | | | | -23.22 | 3.36 | |
| **Hair section 5** | 46.242 | | | | | -23.62 | 3.71 | |
| **Hair section 6** | 46.253 | | | | | -22.42 | 3.34 | |
| **Hair section 7** | 46.264 | | | | | -22.72 | 3.53 | |
| **Hair section 8** | 46.275 | | | | | -23.77 | 3.72 | |
| **Hair section 9** | 46.286 | | | | | -23.08 | 3.26 | |
| **Hair section 10** | 46.296 | | | | | -22.53 | 3.35 | |
| **Hair section 11** | 46.302 | | | | | -23.87 | 3.82 | |

## 2.7 Twycross Zoo Chimpanzee Diet Sheet (2011)

This section presents an archival record from Twycross Zoo of the diet of chimpanzees in 2011, and the subsequent changes to this diet following a review.

Twycross Zoo
The World Primate Centre

Diet Sheet (2011)

# APES

**Species**: **CHIMPANZEE** ***Pan troglodytes***

**Number**: **One adult**

**Breakfast - 8:00am**
100g vegetables (3 types)
100g Greens
150g Mazuri Old World Primate Pellets

**Small random scatter – 9.15-10.30am***
50g popcorn/flaked maize

**Mid morning scatter – 11.30-12.30pm**
500g Browse
100g Greens (3 types)
100g Mazuri High Fibre Primate Sticks**

**Afternoon scatter – 2 - 2.30pm**
500g Browse***
100 g Greens (3 types)
100g Mazuri High Fibre Primate Sticks**

**Summer scatter – 3.30pm***
100g raisins/dried cranberries/mixed nuts/flaked maize (reduce fruit by 100g in 4pm feed)

**Evening – 4pm**
400g fruit (at least 3 types)
250g vegetables (at least 3 types)
100g greens
150g Mazuri Old World Primate Pellets
1 small yoghurt containing 1000mg primrose oil with muesli on top

**Extras**
1 boiled egg every Monday and Friday
Vit D3 oil + Vit E supplement (2mls/day 1000IU/ml), can add to yoghurt

**Fruit options:** apples (can also be offered stewed, without additives), oranges, pears, bananas (no more than 2 per day total), grapes, mango or any other available fruit.
**Vegetable options:** Cauliflower, broccoli, parsnip, carrots, turnips, swede, cabbage
**Greens options:** Courgette, celery, lettuce, tomato, cucumber, pepper, beans, spinach
**Browse options:** Selection of any: poplar, fruit trees (apple, cherry, maple) sycamore, bamboo, willow, beach
**Drinks:** Ad lib water. Weak fruit tea (without milk/sugar) or juice drinks (must be sugar free/'Tooth kind') 250ml maximum twice weekly.

*Can swap food between scatters & use food from rest of diet in scatter feeds.
**Pending sourcing of these sticks (or similar high fibre alternative), use Mazuri Old World Primate pellets in place.
***Swap browse for pellets in winter months, can provide more browse when available.

Changes: Increased proportion of pelleted feed, milk removed, increased browse, reduced sunflower seed, added Vit D3 and Vit E supplement

# Introduction

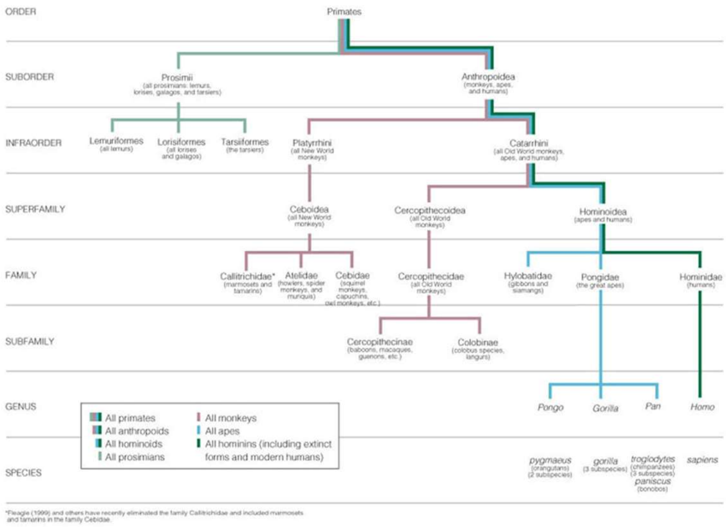


Chimpanzees are members of the Hominadae family with orang-utans and humans. Traditionally there have been two species within the group chimpanzee and these are Pan Troglodytes (the common chimp) and Pan Paniscus (the bonobo). However over the previous years a further division has been made between different groups giving rise to a distinction of a western African group (P. t. verus and P. t. ellioti) and a central/eastern African group (P. t. troglodytes, P. t. schweinfurthii and P. t. marunguensis).

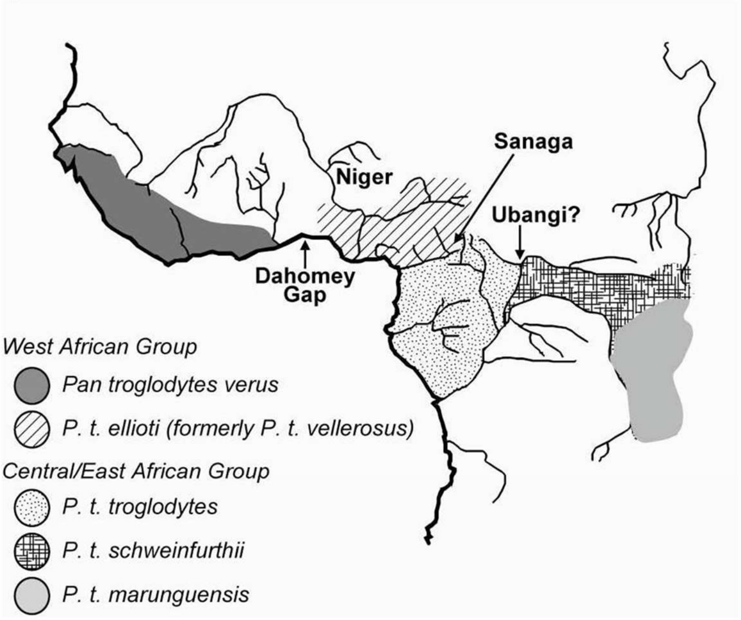


## Social life

Chimp social structure involves a large multi-male and multi-female group within which there is a very clear social hierarchy. An Alpha male is the highest ranking male who controls the group and maintains order during disputes. He will regularly display in order to intimidate other members and ensure his position is secure. The females, however, also have a clear hierarchical structure and this allows them access to resources with higher rank. Their preferred habitat is dense rainforest in west

and central Africa and they are an endangered species due to the destruction of this habitat. Chimpanzees will feed young until around the age of 3 and they reach puberty between 8 and 10. Chimpanzees' rarely live beyond 40 years of age in the wild, but have been known to live beyond 60 in captivity.

**Diet**

In the wild they will spend most of their days eating but shift constantly between different food types even before they are depleted. They are omnivorous but have a staple of fruits, plants, nuts and seeds. Seasonality obviously plays a large part in daily diet and more plants are eaten during the dry season when fruit is not available. Different foods are also preferred at different times of day with fruit being eaten in the morning or evening. Small mammals are taken opportunistically to supplement the diet if available and emerging insects are often exploited.

# Zootrition analysis

As part of the analysis I have fed all the information about the diet into zootrition in order to get a read out of the nutritional constituents. This is based upon the diet fed to Top Chimps on one single day and the weights taken from that day. Some measurements are variable in size, such as handfuls, and this may create variation day to day in the diet.

## Carbohydrate

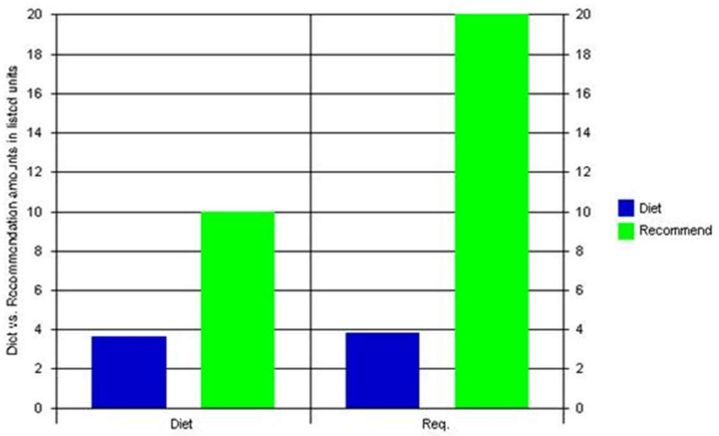


It is obvious from the graph above that the chimps are not getting anywhere near enough non digestible carbohydrate in their diet. Both the ADF (acid detergent fibre) and NDF (neutral detergent fibre) are very low within the current diet, with 57% recommended ADF and only 36% NDF provided.

The majority of ADF and NDF in the current diet is derived from the cauliflower which they currently receive. No browse was included in the dietary analysis as this is understood to be seasonally provided. It would be beneficial to provide a ready supply of forage within the diet in order to increase the cellulose available and more accurately reflect a wild diet. Here feed timing may be useful with fruits being eaten in early morning and evening and forage eaten during the middle of the day.  This could be taken into account for scatter sessions.

## Fats

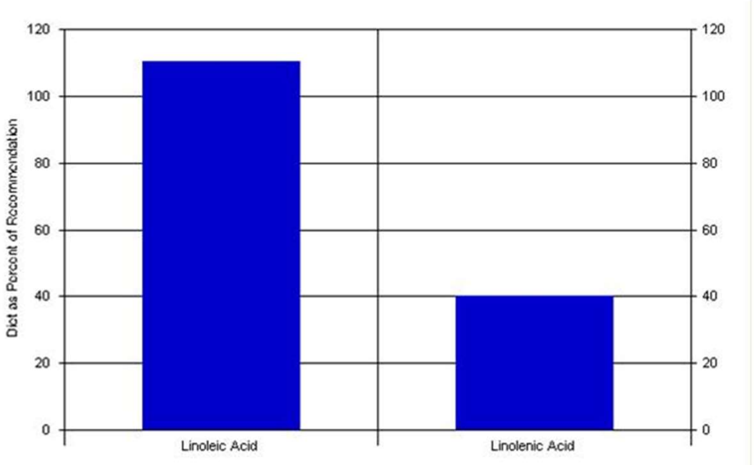


Crude fat made up 6.2% of the analysed diet which is slightly over the recommended level. There is a strong link between obesity and heart disease in chimps therefore it is essential to reduce this. A large proportion of this fat comes from the sunflower seeds (37%) which are very high in fat. The egg does add a little to the fat, however these are required for the energy provided and the protein balance within the diet. Reducing the sunflower seeds with an increase in the primate pellet would counterbalance the drop in protein but reduce the fat present within the diet. Also levels of protein could be maintained through increased feeding of cauliflower and broccoli. The yoghurt in the current ration also adds to the fat content, however this is used as a method of giving the primrose oil supplement. The yoghurt also provides a little extra protein and calcium for the diet.

## Protein

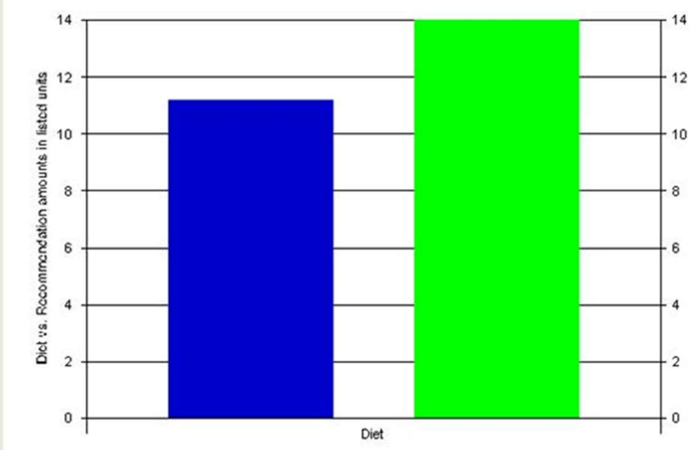


Protein levels within the diet are below recommended levels. The recommended levels found indicated a crude protein measure of 15-22% and the current diet sits at 11.2%. In order to raise this level it would be best to either increase the pelleted feed or to add more cauliflower and broccoli to the ration. Other options include feeding insects as a variable part to the diet for both enrichment and added protein.

## Vitamins and Minerals

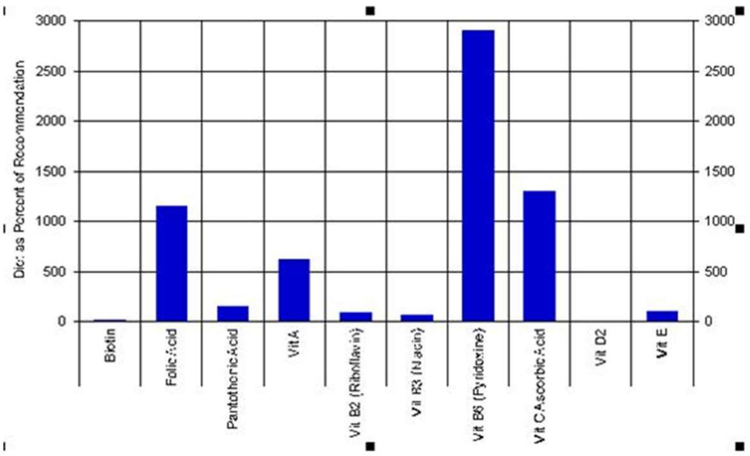


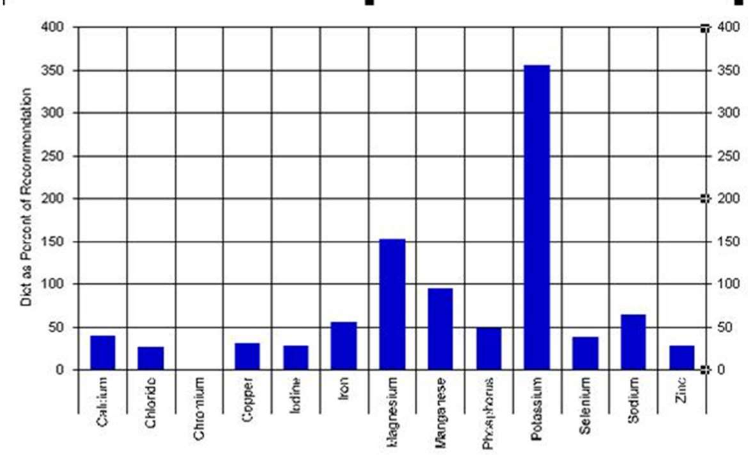


## Biotin

Biotin is necessary for cell growth, the production of fatty acids and the metabolism of fats and amino acids. Biotin levels within the diet are around 15% of what they should be. The sole source for this is through the primate pellets and this is not currently meeting their requirements.

## Vitamin C

The levels of vitamin C are 7-8 times higher than required within the diet. Although this is not a particular problem as excess amounts of Vitamin C are simply excreted I was told that the animals receive a vitamin C supplement in winter months. This is unlikely to be required as there is already plenty of vitamin C available.

## Calcium

Within the current diet calcium represents 0.4% of the dietary intake. This is half of what it should be of 0.8%. 40% of the contribution of calcium comes from the primate pellets and increasing the amount of these fed would also help provide more calcium within the diet. Increasing pellets would also eliminate the requirement for milk which would reduce the risk of R & R.

## Vitamin D3

This is very deficient within the diet, with only 46% of the RDA provided. This could be easily supplemented and would be very beneficial for health.

Pottassium

The potassium within the diet is almost entirely from t

## Trace elements and Minerals

Selenium and copper are particularly deficient within the ration. The most abundant source of these and others within the current ration is within the primate pellets. By far the easiest method for ensuring all trace element requirements are met is to give a higher proportion of pelleted feed. This will help maintain the health within the group.

## Overall recommendations:

The current diet has been working well and this is obvious from the general health of the chimps. The current method of feeding in 4-5 different sessions throughout the day is ideal. However some potential areas for improvement are:

- Reduce the amount of fat within the diet.
    - Reduce the amount of sunflower seeds that are fed each day.
- Increase the fibre content of the diet.
    - Either provide a browse/forage throughout the year or provide a supplement such as Mazuri High Fibre Sticks which could be fed as a mid day scatter.
- Increase the amount of pelleted feed fed daily to around 20% pellets (currently around 4%)
    - This will help with vitamin and mineral balance and help increase the protein content of the diet.
    - New ways of feeding the pellets may have to be devised in order to provide a more enriched experience whilst feeding.
- Feed only around 20% fruit daily (currently 40%)
    - This should be concentrated into the evening feeds to ensure pellets are eaten during the day
- Have fresh browse available daily.
    - To help increase fibre content of diet and provide enrichment.
    - Develop systems for producing browse on site in order to provide a stable supply year round.
- Maybe try weighing out chopped ingredients and using measuring scoops for some ingredients to maintain more similar measures from day to day.
- Increase proportion of vegetables provided to give more low in sugar alternatives to fruit.
- Remove milk and other dairy products from the diet wherever possible.

## Proposed New Diet

This diet has been run through Zootrition. It has addressed the areas identified earlier and satisfies all nutritional requirements.

**Species**: **CHIMPANZEE** ***Pan troglodytes***

**Number**: **One adult**

**Breakfast - 8:00am**
100g vegetables (3 types)
100g Greens
20g sunflower seeds
150g Mazuri Old World Primate Pellets

**Small random scatter – 9.15-10.30am**
50g Pumpkin Seeds

**Mid morning scatter – 11.30-12.30pm**
500g Browse
100g Greens (3 types)
100g Mazuri High Fibre Primate Sticks

**Afternoon scatter – 2 - 2.30pm**
500g Browse
100 g Greens (3 types)
100g Mazuri High Fibre Primate Sticks

**Summer scatter – 3.30pm**
100g Sultanas (reduce fruit by 100g in tea)

**Evening – 4pm**
400g fruit (at least 3 types)
250g vegetables (at least 3 types)
100g greens
150g Mazuri Old World Primate Pellets
1 small yoghurt containing 1000mg primrose oil with muesli on top

**Extras**
1 boiled egg every Monday and Friday
Vit D3 + Vit E supplement (2mls/day 1000IU/ml)

Fruit options: Apples, Oranges, Pears, Bananas (no more than 2 per day total), Grapes, Mango or any other available fruit.
Vegetable options: Cauliflower, Broccoli, Parsnip, Carrots, Turnips, Swede, Cabbage
Greens options: Courgette, celery, lettuce, tomato, cucumber, pepper, beans, spinach
Browse options: Selection of any: poplar, fruit trees (apple, cherry, maple) sycamore, bamboo, willow, beach
Changes: Increased proportion of pelleted feed, milk removed, increased browse, reduced sunflower seed added vit d3 and vit E supplement

## Overall diet info readout from Zootrition

| | | | | | | | | |
|---|---|---|---|---|---|---|---|---|
| Acid Lignin | 0.00 | % | Lactose | - | | Sodium | 0.15 | % |
| ADF | 8.78 | % | Leucine | 0.70 | % | Soluble Protein | - | |
| Arachidonic Acid | 0.00 | % | Lignin | 7.11 | % | Starch | - | |
| Arginine | 0.52 | % | Linoleic Acid | 1.58 | % | STL GLOBAL UPDATES | - | |
| Ash | 7.47 | % | Linolenic Acid | 0.04 | % | Sucrose | - | |
| Beta-carotene | 0.68 | mg/kg | Lysine | 0.44 | % | Sugar | - | |
| Biotin | 0.09 | mg/kg | Magnesium | 0.10 | % | Sulfur | 0.08 | % |
| Bound Protein | - | | Manganese | 35.02 | mg/kg | Taurine | 0.01 | % |
| Calcium | 0.79 | % | ME Carnivore | - | | Threonine | 0.29 | % |
| Cellulose | 0.23 | % | ME Horse | - | | Total Dietary Fiber | 1.87 | % |
| Chloride | 0.19 | % | ME Poultry | - | | Tryptophan | 0.10 | % |
| Cholesterol | - | | ME Primate | 1.67 | kcal/g | Tyrosine | 0.19 | % |
| Choline | 496.73 | mg/kg | ME Ruminant | - | | Valine | 0.45 | % |
| Chromium | - | | ME Swine | - | | Vit A | 28.50 | IU A/g or RE/g |
| Cobalt | 0.18 | mg/kg | Methionine | 0.19 | % | Vit B1 (Thiamin) | 4.76 | mg/kg |
| Copper | 9.39 | mg/kg | Molybdenum | - | | Vit B12 | 9.80 | mcg/g |
| Crude Fat | 5.97 | % | Monounsaturated Fats | 0.77 | % | Vit B2 (Riboflavin) | 4.54 | mg/kg |
| Crude Fiber | 10.48 | % | NDF | 15.52 | % | Vit B3 (Niacin) | 46.63 | mg/kg |
| Crude Protein | 16.56 | % | NFC | - | | Vit B6 (Pyridoxine) | 6.64 | mg/kg |
| Cystine | 0.11 | % | NFE | - | | Vit C Ascorbic Acid | 629.92 | mg/kg |
| Digestible Energy | - | | Nitrogen | - | | Vit D2 | - | |
| Folic Acid | 3.97 | mg/kg | NSC | - | | Vit D3 | 1.14 | IU Vit D3/g |
| Fructose | - | | Pantothenic Acid | 26.31 | mg/kg | Vit E | 68.18 | mg/kg |
| Glucose | - | | Phenylalanine | 0.40 | % | Vit K | 1.04 | mg/kg |
| Gross Energy | 0.67 | kcal/g | Phosphorus | 0.36 | % | Water Soluble Carbohydrates | - | |
| Histidine | 0.13 | % | Potassium | 0.91 | % | WCS GLOBAL UPDATES | - | |
| Iodine | 0.41 | mg/kg | PUFA | 1.30 | % | Zinc | 47.46 | mg/kg |
| Iron | 98.45 | mg/kg | Saturated Fats | 0.55 | % | | | |
| Isoleucine | 0.38 | % | Selenium | 0.10 | mg/kg | | | |

## References


Nutrient requirements of non human primates. Second revised edition, 2003
Crtssey, S., Pribyl, L., 2000. A review of nutritional deficiencies and toxicities in captive New World primates. International Zoo Yearbook 37:355-360.
http://science.jrank.org/pages/1418/Chimpanzees-Eating-habits.html
http://www.mazuri.eu/mediapool/78/786184/data/DATA/29_Mazuri_Primate.pdf
Zootrition V2.6

# References


1. Frost, S. R., Marcus, L. F., Bookstein, F. L., Reddy, D. P. & Delson, E. Cranial allometry, phylogeography, and systematics of large-bodied papionins (primates: Cercopithecinae) inferred from geometric morphometric analysis of landmark data. *Anat. Rec.* **275A**, 1048–1072 (2003).
2. Ventresca Miller, A. *et al.* Sampling and Pretreatment of Tooth Enamel Carbonate for Stable Carbon and Oxygen Isotope Analysis. *JoVE* 58002 (2018) doi:10.3791/58002.
3. Longin, R. New Method of Collagen Extraction for Radiocarbon Dating. *Nature* **230**, 241–242 (1971).
4. Collins, M. J. & Galley, P. Towards an Optimal Method of Archaeological Collagen Extraction: The Influence of pH and Grinding. *Ancient Biomolecules* **2**, 209–223 (1998).
5. Long, E. S., Sweitzer, R. A., Diefenbach, D. R. & Ben-David, M. Controlling for anthropogenically induced atmospheric variation in stable carbon isotope studies. *Oecologia* **146**, 148–156 (2005).
6. Chenery, C. A., Pashley, V., Lamb, A. L., Sloane, H. J. & Evans, J. A. The oxygen isotope relationship between the phosphate and structural carbonate fractions of human bioapatite. *Rapid Comm Mass Spectrometry* **26**, 309–319 (2012).
7. Smith, C. C., Morgan, M. E. & Pilbeam, D. Isotopic ecology and dietary profiles of Liberian chimpanzees. *Journal of Human Evolution* **58**, 43–55 (2010).
8. Oelze, V. M. Reconstructing temporal variation in great ape and other primate diets: A methodological framework for isotope analyses in hair. *American J Primatol* **78**, 1004–1016 (2016).
9. Sillen, A. & Kavanagh, M. Strontium and paleodietary research: A review. *Am. J. Phys. Anthropol.* **25**, 67–90 (1982).
10. Austin, C. *et al.* Barium distributions in teeth reveal early-life dietary transitions in primates. *Nature* **498**, 216–219 (2013).

11. Simpson, R., Cooper, D. M. L., Swanston, T., Coulthard, I. & Varney, T. L. Historical overview and new directions in bioarchaeological trace element analysis: a review. *Archaeol Anthropol Sci* **13**, 24 (2021).
12. Burton, J. H. & Price, T. D. The ratio of barium to strontium as a paleodietary indicator of consumption of marine resources. *Journal of Archaeological Science* **17**, 547–557 (1990).
13. Sponheimer, M., Deruiter, D., Leethorp, J. & Spath, A. Sr/Ca and early hominin diets revisited: new data from modern and fossil tooth enamel. *Journal of Human Evolution* **48**, 147–156 (2005).
14. Szostek, K., Głąb, H. & Pudło, A. The use of strontium and barium analyses for the reconstruction of the diet of the early medieval coastal population of Gdańsk (Poland): A preliminary study. *HOMO* **60**, 359–372 (2009).
15. Threadgold, M. *The Secrets of the Tea Chimps*. (Angel Eye, 2015).
16. Law, G. & Kitchener, A. C. Twenty years of the tiger feeding pole: review and recommendations. *Int. Zoo Yb.* **54**, 174–190 (2020).
17. Species360 Zoological Information Management System (ZIMS). zims.Species360.org (2023).
